# A multi-scale coevolutionary approach to predict interactions between protein domains

**DOI:** 10.1371/journal.pcbi.1006891

**Published:** 2019-10-21

**Authors:** Giancarlo Croce, Thomas Gueudré, Maria Virginia Ruiz Cuevas, Victoria Keidel, Matteo Figliuzzi, Hendrik Szurmant, Martin Weigt

**Affiliations:** 1 Sorbonne Université, CNRS, Institut de Biologie Paris Seine, Biologie computationnelle et quantitative–LCQB, Paris, France; 2 Italian Institute for Genomic Medicine, Torino, Italy; 3 Department of Basic Medical Sciences, College of Osteopathic Medicine of the Pacific, Western University of Health Sciences, Pomona CA, United States of America; University of Illinois at Urbana-Champaign, UNITED STATES

## Abstract

Interacting proteins and protein domains coevolve on multiple scales, from their correlated presence across species, to correlations in amino-acid usage. Genomic databases provide rapidly growing data for variability in genomic protein content and in protein sequences, calling for computational predictions of unknown interactions. We first introduce the concept of *direct phyletic couplings*, based on global statistical models of phylogenetic profiles. They strongly increase the accuracy of predicting pairs of related protein domains beyond simpler correlation-based approaches like phylogenetic profiling (80% vs. 30–50% positives out of the 1000 highest-scoring pairs). Combined with the direct coupling analysis of inter-protein residue-residue coevolution, we provide multi-scale evidence for direct but unknown interaction between protein families. An in-depth discussion shows these to be biologically sensible and directly experimentally testable. Negative phyletic couplings highlight alternative solutions for the same functionality, including documented cases of convergent evolution. Thereby our work proves the strong potential of global statistical modeling approaches to genome-wide coevolutionary analysis, far beyond the established use for individual protein complexes and domain-domain interactions.

## Author summary

Interactions between proteins and their domains are at the basis of almost all biological processes. To complement labor intensive and error-prone experimental approaches to the genome-scale characterization of such interactions, we propose a computational approach based upon rapidly growing protein-sequence databases. To maintain interaction in the course of evolution, proteins and their domains are required to coevolve: evolutionary changes in the interaction partners appear correlated across several scales, from correlated presence-absence patterns of proteins across species, up to correlations in the amino-acid usage. Our approach combines these different scales within a common mathematical-statistical inference framework, which is inspired by the so-called direct coupling analysis. It is able to predict currently unknown, but biologically sensible interaction, and to identify cases of convergent evolution leading to alternative solutions for a common biological task. Thereby our work illustrates the potential of global statistical inference for the genome-scale coevolutionary analysis of interacting proteins and protein domains.

## Introduction

Essential to life at the molecular level is the interplay of molecules and macromolecules. Interactions contribute to diversity and coordination of reactions to accomplish feats that would be impossible if all parts worked fully in isolation. Proteins are no exceptions and many of them undergo concerted interactions to achieve their full potential. Many interactions have been described in detail, including inter- and intra-protein domain-domain interactions, which will be the focus of this work. However, many more meaningful interactions await to be discovered and explored. An issue with the experimental description of such interactions is that many are transient and that high-throughput technologies to identify such interactions are very error prone [1]. Advances in sequencing technology and the subsequent accumulation of vast sequence databases have fueled the generation of mathematical frameworks which aim to identify protein-protein interactions [2, 3]. Some of these techniques rely on the correlated evolution of interacting proteins [4–10]. Whenever interactions are conserved across many organisms, sufficient sequence examples are now in principle available to computationally identify novel interactions relying on sequences alone.

We suggest a statistical approach based on the *coevolution of interacting protein domains*. Coevolution can be detected at very different scales, ranging from the correlated presence or absence of related proteins (or their genes) across genomes, down to the correlated usage of amino-acids in residues, which are located in different proteins but in contact across the interface. Each scale contains valuable information for detecting and understanding interactions between proteins and their domains, and adapted methods have been designed to unveil this information from data. However, none of the scales contains exhaustive information. Therefore, our work proposes a coherent mathematical-algorithmic framework bridging different scales, thereby combining the information content of the different scales.

The first, largest scale concerns the correlated presence and absence of interacting proteins in genomes. If a biological function depends on two proteins simultaneously (not necessarily via their direct physical interaction, but via any functional relation), we will either observe both proteins in a genome, i.e. the function is present, or none of them, i.e. the function is absent. More rarely we may observe the presence of only one of the two proteins. This idea is at the basis of a classical method called *phylogenetic profiling* [4, 5], which uses presence/absence correlations across genomes to predict interactions. Its accuracy suffers, however, from a number of shortcomings and confounding factors:

1. *Phylogenetic relationships* between considered genomes may introduce correlations unrelated to biological function; single evolutionary events may be statistically amplified when closely related species are included in the data. Evolutionary models taking into account the underlying species tree, have been proposed [11–13] to prune such correlations.
2. Correlations may result from direct couplings, e.g., when two domains or proteins interact physically, but they may be caused by intermediate partners: If A co-occurs with B, and B with C, also A and C will show correlations. Analyses based on partial correlations [14] and spectral analysis [15] have been proposed to *disentangle direct from indirect correlations*.
3. Simple presence/absence patterns cannot *discriminate physical interaction from more general relationships*, like co-occurrence in a biological pathway or genomic co-localization. Here, using full amino-acid sequences instead of presence/absence patterns may help to refine the analysis, e.g. via the comparison of protein-specific phylogenetic trees [6].

This last point actually suggests to change resolution, and to consider coevolution at the residue scale to refine the analysis of phylogenetic profiles. The last decade has seen important progress in this respect [16, 17], related to methods like Direct Coupling Analysis (DCA) [18, 19], Gremlin [20] or PsiCov [21]. DCA-type methods were initially developed to capture the correlated amino-acid usage of residues in physical contact. Concerning interacting proteins, they have triggered a breakthrough in using sequence covariation for inter-protein residue-residue contact prediction [16, 17], which in turn is used to guide computational quaternary structure prediction [22–25].

Beyond structure prediction, DCA was suggested for the identification of interacting proteins [9, 10, 26, 27]. Such analysis requires the construction of a large joint multiple-sequence alignment (MSA) of two protein families, with each line of the MSA containing two potentially interacting proteins. However, when proteins possess numerous paralogs inside the same genome, the matching of potentially interacting paralog pairs becomes computationally hard [8, 28]. In some cases, genomic co-localization (e.g. bacterial operons) helps to identify the interacting paralogs [18, 23, 24]. Residue-residue coevolution itself has recently been proposed as a means to match paralogs, and to identify specific interaction partners [26, 27]. While results for individual protein pairs are promising, the computational cost is prohibitive for genome-wide analysis, i.e., for systematically investigating all pairs of present protein families for signatures of coevolution and thus interaction.

Our work addresses this issue, together with Points 2 and 3 given above. We propose a common statistical-modeling framework, which is applied successively to the genomic and the residue scale (presence/absence patterns and amino-acid sequences) of coevolution. It is intended to extract information from data, which cannot be extracted at each individual scale. Performing the genome-wide analysis on the coarse scale of presence/absence patterns, we can identify promising protein-domain pairs, which are subsequently analyzed using DCA at the fine residue scale. A direct comparison of our genome-wide results with those obtained using a phylogeny-aware method [29] unveils some interesting connections between Points 1 and 2 above.

## Results

### Phyletic couplings improve the prediction of domain-domain relationships beyond correlations

**Fig 1:**
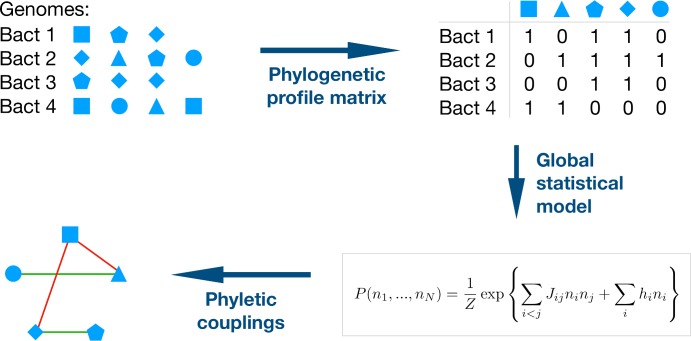
Schematic representation of the inference of phyletic couplings. **–**The composition of bacterial genomes in terms of protein families is extracted from the Pfam database. The presence and absence of each family is coded into the binary phylogenetic profile matrix (PPM); note that this matrix does not account for the presence of multiple paralogs of a domain. The statistics of the PPM is reproduced by a global statistical model *P*(*n*_*1*_, …,*n*_*N*_) for a full genomic phylogenetic profile, the model corresponds to a lattice gas model in statistical physics. The strongest positive couplings (favored domain-domain co-occurrence) are expected to stand for positive relationships between domains, like domain-domain interactions or genomic co-localization. Negative couplings (avoided co-occurrence) is expected to indicate alternative solutions for the same biological function, like in cases of domain families in a common Pfam clan, or for convergent evolution.

The analysis starts with a fairly standard construction of phylogenetic profiles [5], as outlined in Fig 1. Multiple-sequence alignments are needed at a later stage to perform inter-protein DCA. Since Pfam MSA have been extensively used in this respect, the analysis is performed on the domain level [30], using Pfam [31] as the input database. Pfam is based on reference genomes and we use the 1041 bacterial ones. The bacterial model organism *Escherichia coli* is used as a reference, i.e. only the 2682 domain families existing inside the K12 strain of *E*. *coli* are considered (the *Supplement* S1 Text Fig K shows that the results are robust when expanded beyond this choice). Since our method is based on covariation of presence and absence of domains in genomes, only variable domains existing in at least 5% and at most 95% of the considered genomes are considered, leaving 2041 domains. Note that the upper limit removes domains, which are omnipresent in the bacteria–mostly related to central life processes like replication, transcription and translation. However, being omnipresent, these domains cannot give any covariation signal within phylogenetic profiling. They could be analyzed using the finer residue-scale of coevolution, which might bring complementary evidence for interactions between these domains, but this analysis is out of scope in the current paper. The final input data are given by a binary phylogenetic profile matrix (PPM) of *M* = 1041 rows (species) and *N* = 2041 columns (domains), with entries 1 if a domain is present at least once in a genome, and zero if it is absent, cf. *Methods* and Fig 1.

**Fig 2:**
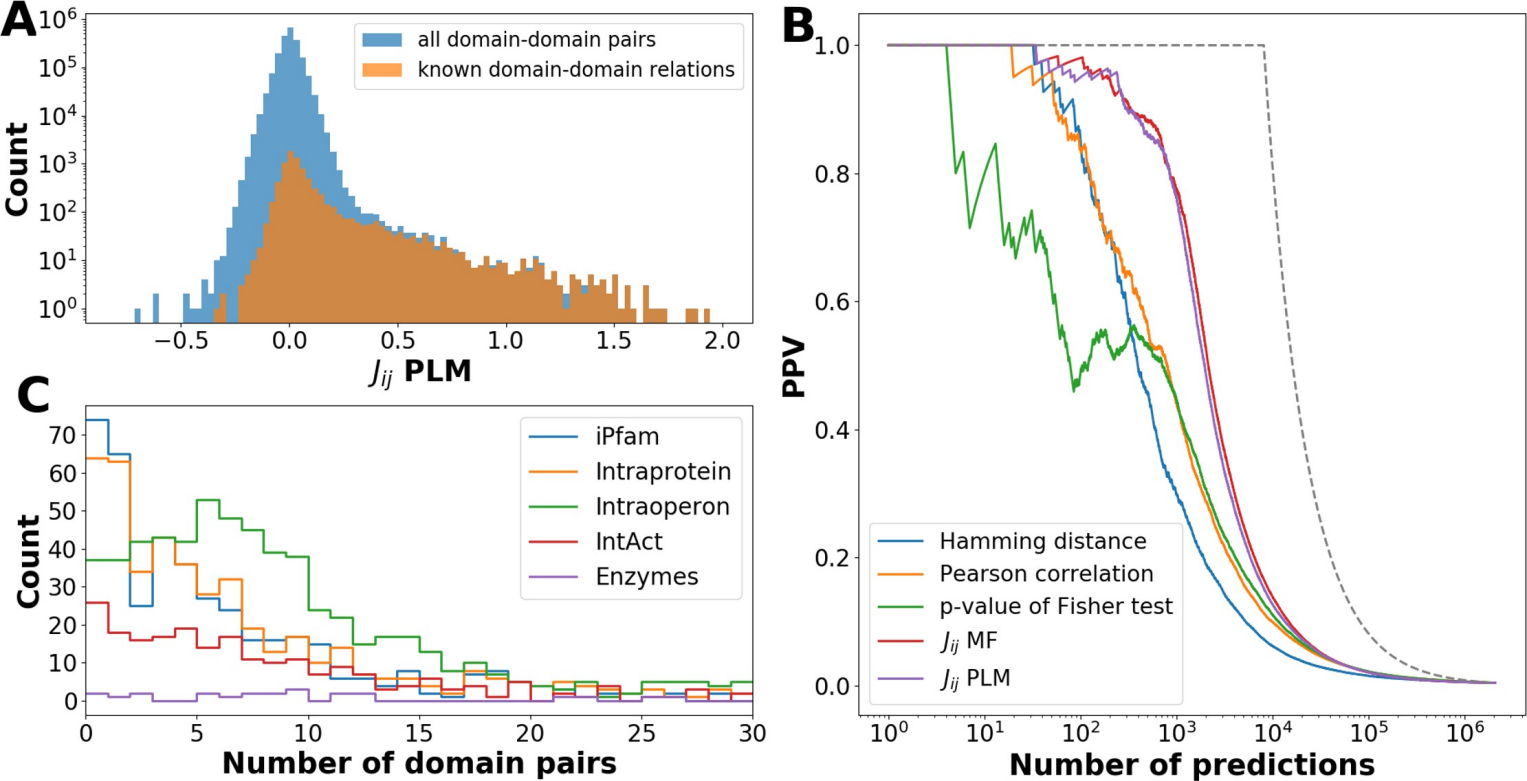
Phylogenetic couplings predict domain-domain relationships. **–***Panel A* shows histograms of couplings *J*_*ij*_ as inferred using pseudo-likelihood maximization (PLM), cf. *Methods*, for all domain-domain pairs (blue) and for the subset of known positive domain-domain relations (brown). The histogram shows a dominant central peak around zero (note the logarithmic scale of the counts) with a pronounced fat tail for positive couplings. In contrast to the central peak, this tail is strongly dominated by the known positive domain-domain relations. A small tail for negative couplings is visible, too, but much less pronounced. *Panel B* shows the PPV (positive predictive value), defined as the fraction of known domain-domain relations in between the strongest couplings or correlations. A random prediction would correspond to a flat line close to zero; a perfect prediction would follow the dashed black line. Note that the curves corresponding to phylogenetic couplings (inference vis PLM (pseudo-likelihood maximization) or MF (mean field), cf. *Methods*) are substantially higher than those using correlation measures. *Panel C* shows, in bins of 100 domain pairs ordered by their phyletic couplings, the number of pairs belonging to the different parts of the positive-relation list (note that the categories are not exclusive, so the sum of different categories may exceed 100). We find enrichment of co-localized and interacting domain pairs, but not of related enzymes.

An important breakthrough in coevolutionary analysis at the residue level was the step from a local correlation analysis to global maximum-entropy models [16, 32], which are able to disentangle indirect (i.e. collective) effects in correlations, and to explain them by a network of direct couplings. Here we show that the same idea can be adapted to phylogenetic profiling, and leads to a strongly increased accuracy in predicting relationships between domains. The method, which we call *Phyletic Direct Coupling Analysis (PhyDCA)*, infers a statistical model *P*(*n*_*1*_, …,*n*_*N*_) for the phylogenetic profile of an entire species, i.e. for a binary vector (*n*_*1*_, …,*n*_*N*_) signaling the presence or absence of all *N* considered domains in the considered species, cf. *Methods* for details. The PhyDCA model resembles a *lattice-gas model* in statistical physics, describing *N* coupled particles that can be present or absent. The phyletic coupling *J*_*ij*_ between particles / domains *i* and *j* can be positive–i.e. the presence of one domain favors the presence of the other. In this case we expect a positive relationship between the two domains, corresponding to biological processes requiring both domains. The coupling *J*_*ij*_ can also be negative–i.e. the presence of one domain favors the absence of the other. We would expect that these domains have overlapping functionalities, and one of the two is sufficient to guarantee this functionality in a species. Fig 2A shows a histogram of the couplings found for the phylogenetic coupling matrix. We observe a clear bulk of small coupling values concentrated around zero, with a broad tail for larger positive values, and a less pronounced tail for negative values.

The performance of PhyDCA can be assessed by comparing the domain pairs of strongest phyletic couplings to a carefully compiled list of 8,091 known domain-domain relations. As is explained in *Methods*, we have included genomic, functional and structural relationships: domains may coexist inside a single protein, they may be co-localized in an operon, they may be in contact in an experimental crystallographic structure or an interaction might be known according to other experimental techniques, or they may belong to enzymes catalyzing related reactions.

The PhyDCA couplings *J*_*ij*_ are ordered by size, and the fraction of positive relations in between the highest-scoring domain-domain pairs is calculated (PPV = positive predictive value). Fig 2B shows the results: we observe a strong enrichment in known positive relations in between strongly phyletically coupled domain-domain pairs. This enrichment is much stronger than for local correlation measures like Hamming distance, Pearson correlation or p-value of Fisher’s exact test applied individually to two domains (i.e. two columns of the PPM): E.g., for the first 1000 predictions we observed a PPV of about 0.8 for the phyletic couplings, and only 0.3–0.5 for the different correlation measures. Interestingly, the difference between applied PhyDCA approximations based on mean-field or pseudo-likelihood maximization is much smaller than expected from experience with contact-prediction in standard DCA. As is shown in the *Supplement* S1 Text, Fig C, couplings of both approximations are highly correlated (Pearson correlation 93% for all domain pairs, 97.5% for the known positives), resulting rather in a minor relative reranking of the two predictions than in a different accuracy. Similarly, the effect of applying the average-product correction (cf. *Methods*) has only a limited effect. As is shown in Fig 2C, interacting and co-localized domain pairs are enriched in the predictions of large positive couplings, whereas enzymes from related metabolic reactions are not. Interestingly, pairs with intra-protein co-localization are most enriched in between the strongest PhyDCA couplings (the comparable iPfam enrichment can be traced back to intra-chain co-crystals, i.e., to the same signal), which is confirming their evident functional relationship as compared to, e.g., pairs in distinct proteins coded in a joint operon. However, even inside multi-domain proteins the coupling density remains low, which results from both the sparsity of strong couplings in general, and the fact that the same domain may exist in very different protein architectures, thereby reducing correlation signals related to a specific multi-domain architecture.

From an overlay of the *J*_*ij*_-histograms for all domain pairs and those with known relations in Fig 2A, we immediately see that the fat tail is strongly dominated by the known relations. This domination stops as we leave the tail and enter the bulk of the histogram, as a result we can determine a threshold of 0.3–0.5 for couplings to be significant. This threshold is coherent with the sharp drop in PPV in Fig 2B after about the first 1000 predictions.

Databases of genome-wide protein-protein or domain-domain interactions are currently incomplete. We therefore expect the real PPV to be even higher than the one measured in Fig 2: strongly coupled domain-domain pairs *not* belonging to our list of positives may actually be considered as predictions for new, currently unknown relations. According to the observations in Fig 2C, these relations might be direct physical interactions, but also genomic co-localization (frequently related to joint biological function). Before exploring these possibilities in more detail and on the finer scale of the residue-residue coevolution, we compare the PhyDCA results to phylogeny-aware correlation analysis and investigate the negative tail of the *J*_*ij*_ distribution.

### Comparison of phyletic couplings to phylogeny-aware analysis of correlated presence/absence patterns

Phyletic couplings are, like simpler correlation measures, based on counting co-presence and co-absence of proteins or domains. However, due to the uneven phylogenetic distribution of species in our dataset, single evolutionary event may be amplified when appearing in an ancestor of several closely related species. More importantly in the context of this study, phylogeny may introduce spurious correlations in the presence and absence of domains, which are not related to biological function.

To remove this bias, several methods have been proposed, cf. [11, 13], which use evolutionary models to decide, if observed correlations can be explained by phylogeny alone (i.e. by independent evolution on a phylogenetic tree), or remain significant even when such phylogenetic effects are removed. Since this idea is complementary to the one behind PhyDCA, it is important to compare the outcome of both approaches.

To this end, we have used the CoPAP (coevolution of presence-absence patterns) server [29]. It uses the same type of binary input matrix of our approach, and is able to efficiently treat matrices of more than 2,000 domains across more than 1,000 species. As an output, CoPAP provides p-values measuring the significance of correlated domain presence and absence, as compared to independently evolving domains on the same phylogenetic tree. The group of maximum significance (*log*_10_*p* < −7.9) contains 3,611 domain pairs, out of which 1,251 (34.6%) are true positives in our list of known domain-domain relationships.

**Fig 3:**
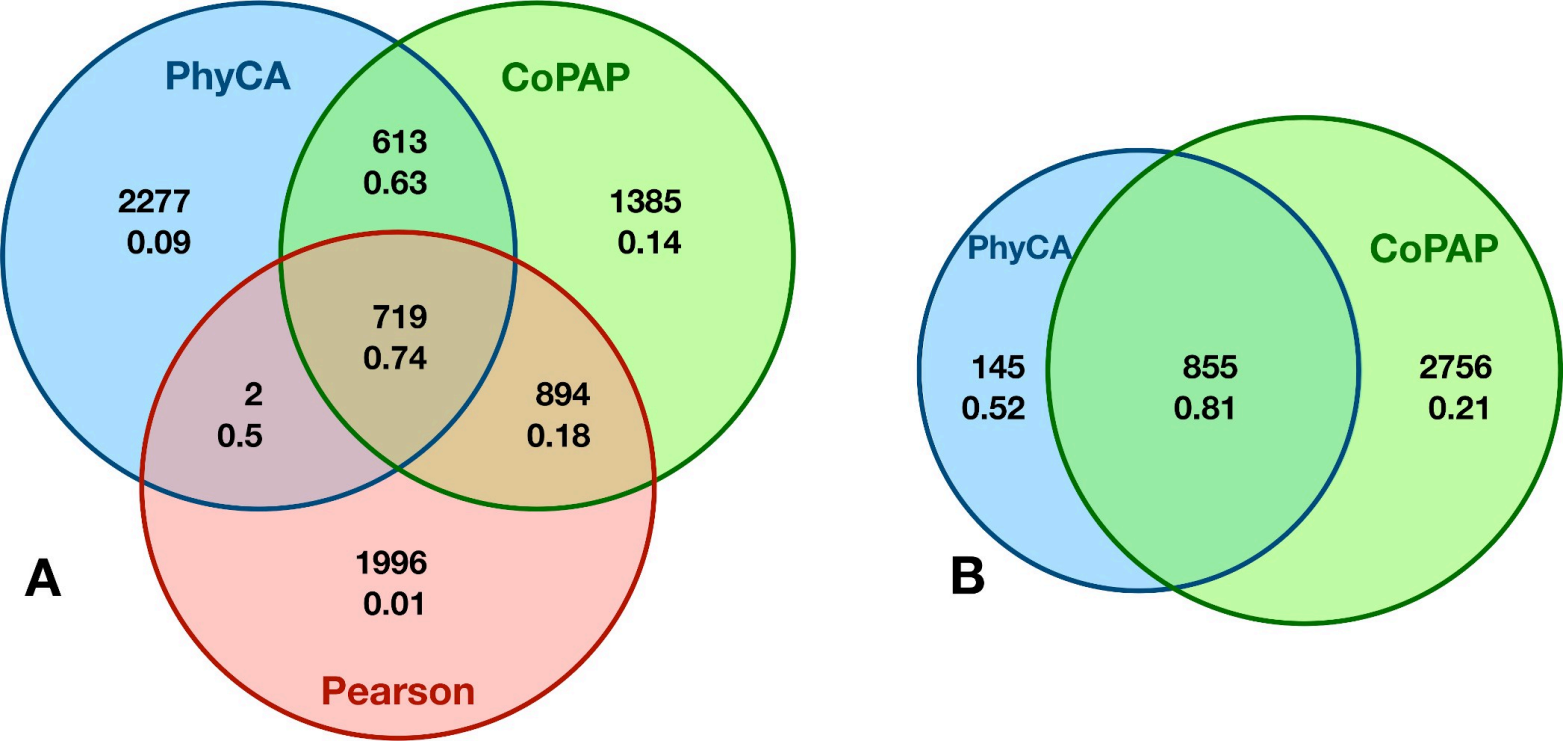
Comparison of simple correlations, phyletic couplings and phylogeny-corrected correlations. **–**Panel A shows a Venn diagram for the 3,611 first predictions of each of the three coevolution measures as extracted by Pearson correlation (red), PhyDCA (blue) and CoPAP (green). Numbers are the size of the corresponding intersection, and the PPV indicating the fraction of true positives according to our list of positive domain-domain interactions. Panel B compares the first 3,611 CoPAP predictions of highest possible significance, with the most significant 1,000 PhyDCA predictions. Most of them (855) are found to be significant by CoPAP, and of very high PPV (81%). However, not all CoPAP pairs are strongly coupled, and thus PPV is reduced (21%).

Since a further sorting of these pairs using CoPAP results is not possible (p-values are calculated using finite simulations), we compare them to the first 3,611 domain pairs extracted by PhyDCA, and to the 3,611 domain pairs of highest Pearson correlation. The Venn diagram in Fig 3 and the numbers given in Table 1 allow for a number of interesting observations:

- While CoPAP and PhyDCA have similar global PPV, with an advantage for CoPAP (34.6%) over PhyDCA (31.2%), Pearson correlation performs substantially worse (PPV 19.7%).
- Very small fractions of the correlated pairs, which are discarded by PhyDCA or CoPAP, are TP: PhyDCA discards 2,890 pairs of PPV 6%; CoPAP discards only 1,998 pairs, but with even lower PPV (1.2%).
- 74% of the 721 correlated pairs, which are retained by PhyDCA, are TP. Note that almost all of them (719/721) show also a significant CoPAP signal.
- Only 43% of the correlated pairs, which are retained by CoPAP, are TP. PhyDCA divides them into two groups of comparable size but distinct PPV. For the 719 pairs retained also by PhyDCA, the PPV rises to 74%. The other 894 pairs have weak phyletic couplings, so their significant correlation has to be interpreted as dominated by indirect effects. Actually only 18% are TP.
- When going to lower Pearson correlations, both CoPAP and PhyDCA decrease their accuracy. However, their intersection shows 613 pairs with a high PPV of 63%.
- The 2,277 pairs only identified by PhyDCA have a low PPV of only 9%. This is coherent with Fig 2B, which shows a sharp PPV drop in PhyDCA after the first ca. 1,000 phyletic couplings. We have therefore compared these 1,000 domain pairs separately to CoPAP. A vast majority of 855 pairs have the highest possible significance in CoPAP, this intersection has a PPV of 81%. The other 15% have lower CoPAP scores and lower PPV (52%). Interestingly, only 21% of the 2,756 strongest CoPAP without strong coupling are TP, illustrating again the capacity of PhyDCA to–at least partially–disentangle direct couplings from indirect correlations.

**Table 1:** Comparison of the predictions of Pearson correlation, PhyDCA and CoPAP. –We analyze the different combinations between the 3611 highest scoring predictions according to each of the three scores. In the first three columns, “YES” means that predictions are retained for the concerned score, “NO” means that predictions are discarded by the score, and “–” indicates, that the score is not taken into account.

Pearson	PhyDCA	CoPAP	Elements	TP	PPV
–	–	YES	3611	1251	0.346
–	YES	–	3611	1126	0.312
–	YES	YES	1332	915	0.687
–	YES	NO	2279	211	0.093
–	NO	YES	2279	346	0.152
YES	–	–	3611	713	0.197
YES	–	YES	1613	689	0.427
YES	–	NO	1998	24	0.002
YES	YES	–	721	531	0.736
YES	YES	YES	719	530	0.737
YES	YES	NO	2	1	0.500
YES	NO	–	2890	182	0.063
YES	NO	YES	894	159	0.178
YES	NO	NO	1996	23	0.012
NO	–	YES	1998	572	0.286
NO	YES	–	2890	595	0.206
NO	YES	YES	613	385	0.628
NO	YES	NO	2277	210	0.092
NO	NO	YES	1385	187	0.135

In principle, CoPAP and PhyDCA treat very different confounding factors of coevolutionary analysis–phylogenetic biases and indirect correlations. So, it might appear astonishing that almost none of the correlated pairs, which are strongly coupled in PhyDCA, are actually discarded by CoPAP. The reason might be given by the spectral properties of the covariance matrices of the input data, and their relation to phylogeny and direct couplings. As shown in [33], the phylogenetic bias is most evident in the largest eigenvalues of the data-covariance matrix. These correspond mostly to extended eigenmodes, which in turn give rise to a dense network of small couplings [15, 34]. On the contrary, the strongest pairwise couplings are related to small eigenvalues with more localized eigenmodes, which give rise to strong, sparse couplings. Phylogenetic biases and strong direct couplings are thus related to different tails of the eigenvalue spectrum of the covariance matrix, the strongest PhyDCA couplings are thus robust with respect to phylogenetic biases.

On the other hand, we expect non-phylogenetic but indirect correlations to exist, related to the observation that PhyDCA separates the CoPAP output into strongly coupled pairs of high PPV, and weakly coupled pairs of reduced PPV. To further illuminate these indirect effects, we have introduced Fig H into the *Supplement* S1 Text, which shows a scatter of phyletic couplings vs. Pearson correlations for the CoPAP output. We find a clear triangular shape of this scatter plot: large couplings imply large correlations, but large correlations exist also between pairs of small coupling. The coupling network is thus sparser than the correlation network. Since the PhyDCA model reproduces all correlations, at least some of them must be induced indirectly. We have also taken the network of the before-mentioned 1,000 strongest phyletic couplings, and studied the correlations as a function of the distance along this network. As is shown again in the *Supplement* S1 Text, Fig I, the strongest correlations appear between directly coupled pairs, and the correlations decay with distance until they saturate at a low but non-zero level. This observation is coherent with the idea, that the empirical correlations found in the data have at least three contributions–direct correlations induced by direct couplings (at distance 1), indirect couplings induced by coupling chains, and a ground level of correlations, which possibly result from phylogenetic correlations between the species. Taking alternatively the network induced by the 1,613 domain pairs of high Pearson correlation and CoPAP score, we find a slower decay of correlations along the network, cf. Fig J in S1 Text. At same distance, pairs on the phyletic coupling network are less correlated than those on the correlation/CoPAP network, demonstrating that the coupling network more parsimoniously explains the connectivity patterns present in the data.

### Negative phyletic couplings appear between alternative solutions for the same biological function, including cases of convergent evolution

A smaller tail of negative phyletic couplings can be observed in Fig 2A. A negative coupling disfavors the joint presence of two domains in the same genome, i.e., if one of the negatively coupled domains is present in a genome, the other is less likely to be simultaneously present. Intuitively this suggests similar functionalities, one of the two domains is sufficient, the joint presence unnecessary or even costly for a bacterium. Such pairs, called anti-correlogs in [14] were used in [35] to identify analogous enzymes replacing missing homologs in biochemical pathways.

When using *E*. *coli* as a reference genome, the number of such negative couplings is limited, since only domain pairs co-occurring in *E*. *coli* are analyzed. To better understand the meaning of negative couplings, we have therefore extended the original analysis to all 9,358 families containing bacterial protein domains. While results restricted a posteriori to *E*. *coli* are very robust (96% correlation, cf. *Supplement* S1 Text, Fig K), the extended analysis leads to a substantially higher number of negative couplings.

To explore these in some detail, we analyzed the 20 domain pairs with the strongest negative couplings, cf. Table 2 (an extended list is given in Table C in *Supplement* S1 Text). From their detailed analysis it is evident that protein pairs can be classified into three distinct groups. First, we find several cases of convergent evolution as evidenced by proteins with the same or similar activities but distinct protein structures (rankings 1, 2, 9, 14, 15, 16). Second, we find domain pairs of the same fold and, where known, of similar activity. For various reasons these are not described by the same Pfam HMM (rankings 3, 4, 6, 7,8, 10,11, 17,19), but typically belong to the same Pfam clan indicating distant homology. Lastly, there are several cases of relatively unknown activity, and some domains have no known structure (rankings 5, 12, 13, 18, 20).

**Table 2:** The 20 domain pairs of top negative phyletic couplings.

	Pfam 1	Pfam 2	*J*_*IJ*_	Domain 1 description	Domain 2 description
**1**	PF00303	PF02511	-0,9978	Thymidylate synthase	Thymidylate synthase complementing protein
**2**	PF01220	PF01487	-0,9277	Dehydroquinase class II	Type I 3-dehydroquinase
**3**	PF02834	PF13563	-0,9075	LigT like Phosphoesterase	2'-5' RNA ligase superfamily
**4**	PF00406	PF13207	-0,8258	Adenylate kinase	AAA domain
**5**	PF01205	PF02594	-0,7077	Uncharacterized protein family UPF0029	Uncharacterised ACR, YggU family COG1872
**6**	PF13623	PF13624	-0,7051	SurA N-terminal domain	SurA N-terminal domain
**7**	PF04816	PF12847	-0,6316	tRNA (adenine(22)-N(1))-methyltransferase	Methyltransferase domain
**8**	PF00636	PF14622	-0,6281	Ribonuclease III domain	Ribonuclease-III-like
**9**	PF00186	PF02511	-0,6281	Dihydrofolate reductase	Thymidylate synthase complementing protein
**10**	PF01227	PF02649	-0,6118	GTP cyclohydrolase I	Type I GTP cyclohydrolase folE2
**11**	PF06745	PF13481	-0,5844	KaiC	AAA domain
**12**	PF02677	PF08331	-0,581	Uncharacterized BCR, COG1636	Domain of unknown function (DUF1730)
**13**	PF02696	PF03190	-0,5651	Uncharacterized ACR, YdiU/UPF0061 family	Protein of unknown function, DUF255
**14**	PF00311	PF02436	-0,5432	Phosphoenolpyruvate carboxylase	Conserved carboxylase domain
**15**	PF02502	PF06026	-0,5371	Ribose/Galactose Isomerase	Ribose 5-phosphate isomerase A (phosphoriboisomerase A)
**16**	PF00245	PF05787	-0,5333	Alkaline phosphatase	Bacterial protein of unknown function (DUF839)
**17**	PF00075	PF13456	-0,5317	RNase H	Reverse transcriptase-like
**18**	PF01169	PF02659	-0,5294	Uncharacterized protein family UPF0016	Putative manganese efflux pump
**19**	PF01321	PF05195	-0,5165	Creatinase/Prolidase N-terminal domain	Aminopeptidase P, N-terminal domain
**20**	PF02594	PF09186	-0,5139	Uncharacterised ACR, YggU family COG1872	Domain of unknown function (DUF1949)

Cases of convergent evolution include PF00303 and PF02511, which describe two different thymidylate synthases, the former a 5,10-methylenetetrahydrofolate, the latter a flavin dependent enzyme [36]. Interestingly, PF00186, dihydrofolate reductase is also strongly negatively coupled with PF02511 (but positively to PF00303), since the former is not needed to regenerate 5,10-methylenetetrahydrofolate when the flavin-dependent enzyme is used. Other cases of convergent evolution are PF01220 and PF01487 that describe two classes of dehydroquinases with similar activity but significantly different primary and secondary structure [37]. PF00311 and PF02436 describe proteins in oxaloacetate biogenesis, the former from phosphoenolpyruvate, the later from pyruvate and ATP. PF00245 and PF05787 describe two classes of bacterial alkaline phosphatases, termed PhoA and PhoX with distinct protein folds [38]. PF02502 and PF02436 distinguish two classes of ribose- or phosphoribo-isomerases with differing enzyme folds.

Structurally similar proteins that are identified by different Pfam families are of less interest and will not be separately described. The fact that they are distinct enough in sequence to be covered by separate Pfam families suggests a level of divergent evolution, i.e. one or the other domain has distinct features such us additional interaction partner, distinct activity regulation etc.

Of special interest are domain pairs with unknown function. Ideally, if the function of one Pfam family becomes available one can infer the function of the other family as well. In addition, the evolutionary importance of a given protein family and its activity is often judged by its conservation across different phyla and organisms. This however neglects cases of unknown convergent evolution. Among the highest negatively coupled pairs, we did not find any, where the function of one has been clearly identified and the function of the other has not. However, there are several instances, where a potential role has been loosely associated with one or the other domain. For instance, PF01205 and PF09186 have been suggested to be involved in countering translation inhibition under starvation conditions [39]. These domains are strongly negatively coupled with PF02594, suggesting that the latter might also serve a role in countering translation inhibition. PF01169 and PF02659 are both putative transporters, the former for calcium [40], the latter for manganese ions [41]. Their coupling suggests overlapping specificities or roles. PF02677 and PF08331 describe two entirely unstudied bacterial proteins. The later appears associated with iron-sulfur cluster domains, suggesting a potential role in redox regulation. Lastly, we find a negative coupling between domains PF02696 and PF03190. Both proteins are entirely unstudied in bacteria, but they are also common in Eukaryotes where the latter is a proposed redox protein that has been implicated in fertility regulation in mammals [42]. It would be interesting to unveil their function in the bacteria.

It might be interesting to study the context, in which these negative couplings appear in the PhyDCA network. To this end we have taken all couplings of absolute value above 0.3, resulting in a sparse network of 82 negative, and 3173 positive links. We have now studied the triangles in the resulting network, which have at least one negative coupling. From the fact, that positive links are close to 40times more frequent than negative links, we would expect the other two links of the triangle to be typically positive. On the contrary, we do find only triangles with exactly two negative and one positive coupling; they contain 29 out of the 82 negative phyletic couplings. No so-called “frustrated” triangles are found, where both supplementary links in the triangle are positive. This indicates that more likely entire processes are realized by alternative solutions, than single domains are exchanged against each other within an otherwise positively correlated solution.

### A residue-scale DCA analysis of phylogenetically coupled domain pairs unveils directly coevolving pairs

As seen in Fig 2C, a large positive phyletic coupling is a strong signal for a positive relationship between two domains, but not necessarily for a direct physical interaction of the two domains within a protein complex. Furthermore, co-localization of two domains either inside the same protein (i.e. an evolutionary conserved protein architecture) or inside the same operon may lead to strong phyletic couplings.

Relying only on the coarse scale of coupled presence and absence in genomes, does not reveal more detailed information. Since the number of domain-domain pairs under question is limited as compared to all domain pairs existing in *E*. *coli*, we can afford computationally more expensive approaches, which study coevolution of domain pairs at the individual residue scale. To this effect, we use the procedure suggested in Gueudré et al. [27]: Two Pfam MSA for the two domain families are matched using a variant of DCA such that (a) only sequences appearing inside the same species are matched and (b) the inter-domain covariation as measurable by DCA is maximized. In [27] it was shown that this idea allows to identify protein-protein interactions via a large coevolutionary score between the two domains at a sufficiently large joint MSA. DCA scores above 0.2 at an effective sequence-pair number of at least 200 (sequences below 80% sequence identity, cf. *Supplement*) can be considered as a strong indicator for a potential interaction [10, 27]. On the contrary, according to [43], a low DCA score is not necessarily a sign for the absence of a physical interaction. A low score might also originate from a relatively small or structurally not well-conserved interface, both resulting in a weak coevolutionary signal.

**Fig 4:**
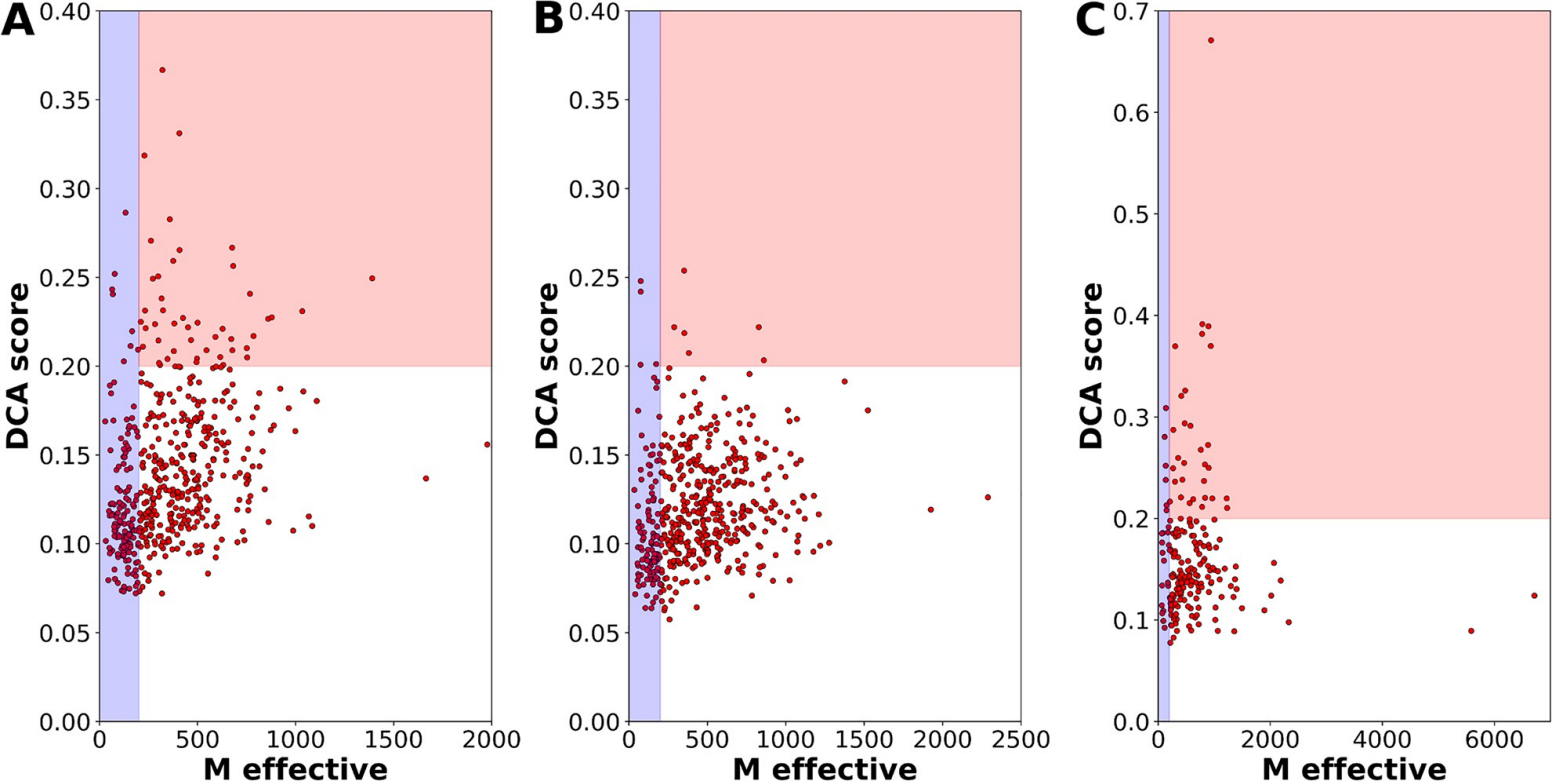
DCA identifies strong residue-scale coevolution between phyletically coupled domain pairs. –Panel A shows the effective sequence number (defined as the sequence number at 80% maximum sequence identity, cf. *Supplement* for the precise definition) and the DCA scores for the 500 domain pairs of strongest phyletic coupling not belonging to the positive-relation set (i.e. the 500 most significant predictions). The interesting region is the red one, where sequence numbers are sufficient to provide reliable DCA results, and DCA scores are beyond 0.2 as established in [10]. Panel B shows, as a comparison, the results for 500 randomly selected domain pairs. Only very few pairs show substantial scores, most of them related to very short peptides. Panel C shows a positive control, the 200 pairs of highest phylogenetic couplings belonging to iPfam are analyzed analogously. The fraction and the amplitude of high DCA scores is slightly increased with respect to Panel A, but the qualitative behavior is similar.

We have applied the progressive paralog matching procedure of [27] to the 500 most strongly coupled domain pairs, which are not in our previously constructed test set of positive domain-domain relations, i.e. to the first 500 *prediction*s at the scale of phyletic couplings. The results are presented in Fig 4A: 360 domain pairs have an *M*_*eff*_ above 200, and DCA results can thus be considered reliable. Of those 45 pairs have an inter-domain DCA score above 0.2 (24 out of the first 200 PhyDCA predictions). This number is significantly larger than for randomly selected protein pairs, cf. Fig 4B: only 10 pairs have a score above 0.2 and *M*_*eff*_ above 200, mostly related to short amino-acid sequences. This shows that the preselection by high phylogenetic couplings leads to a subsequent enrichment of high DCA scores also at the residue scale. For comparison, we have also applied the matching procedure to the 200 domain-domain pairs, which are known to interact by iPfam [44], and which have high phylogenetic couplings, cf. Fig 4C. 29 have a significant DCA score at large enough sequence number. Interestingly, the signal is only marginally stronger than for the newly predicted relations, which are discussed in more detail below. In Fig G the *Supplement* S1 Text, we analyze also the 200 phyletically most positively coupled domain-domain pairs, which co-occur inside the same protein in *E*. *coli*. In their case, the DCA score is found to be substantially larger. This is to be expected, since due to the intra-protein co-localization no paralog-matching has to be applied, and therefore the joint MSA of the two domain families are expected to be of higher quality. However, also in this case, some pairs show a low DCA score despite a large sequence number. This is to be expected, since not all domain-domain pairs inside a multi-domain protein have physical interactions, and also small and structurally non-conserved interfaces may lack clear DCA signals, cf. [43].

### Many predictions of domain-domain interactions resulting from PhyDCA and residue-level DCA are biologically interpretable

Domain pairs with both strong PhyDCA and residue-level DCA signals are our strongest candidates for predicted domain-domain interactions. Since they are not co-localized in the same protein, they also provide predictions for new protein-protein interactions. We analyze here in detail the 24 pairs with a DCA score larger than 0.2, which result from the first 200 PhyDCA predictions.

Among these 24 pairs we find several examples of known interactions that have not yet been structurally resolved. These include K^+^ transporter subunits KdpC (PF02669) and KdpA (PF03814) [45], Sigma54 activator (PF00158) and Sigma54 activator interacting domain (PF00309) [46] and exonuclease VII subunits domains PF02609, PF2601 and PF13742 [47].

For several additional positively coupled pairs an interaction seems functionally very likely but to our knowledge no interaction studies are available. These are all proteins involved in pilus formation or maturation. Domain PF06750 is a putative methyl transferase domain in the prepilin peptidase PppA, and proposed to interact with methylation motif domain PF07963, found in numerous pilin proteins and with PF05157, a type II secretion system protein [48, 49]. PF05157 is also predicted to interact with domain PF05137 found in the PilN fimbrial assembly protein required for mating in liquid culture [50].

Of interest, there are predicted interactions for several members of biosynthetic pathways catalyzing either consecutive or closely following reactions. These include domains PF02542 and PF13288 of isoprenoid biosynthesis enzymes Dxr and IspF, domains PF00885 and PF00926 of riboflavin biosynthesis enzymes RisB and RibB and domains PF01227 and PF01288 of tetrahydrofolate biosynthesis enzymes Gch1 and HppK. A more complex connection is predicted between multiple domains of molybdenum cofactor biosynthesis enzyme MoaC (PF01967), MoeA (PF03453 and PF03454) and MoaA (PF06463). Similarly, scores suggest a protein-protein interaction between domains of hydrogenase maturation enzymes HypF (PF07503) with HybG (PF01455) and HycI (PF01750).

Perhaps most intriguing are the observation of strongly coupled co-occurrence and potential protein-protein interactions of two proteins pairs. Ada (PF02805) and AlkA (PF06029) are two enzymes involved in DNA repair in response to alkylation damage [51, 52]. One of the proteins serves as demethylase of guanosyl residues whereas the other excises alkylated nucleotides. These seemingly complementary functions suggest that an interaction is plausible. The other pair is YoeB (PF06769) with HicA (PF07927). These two proteins constitute two toxins of distinct toxin-antitoxin systems. Both proteins inhibit translation by distinct and complementary mechanisms and an interaction seems plausible. YoeB blocks the ribosome A site leading to mRNA cleavage [53]. HicA interacts with mRNA directly and thus acts independent of the translation apparatus [54].

Additional and perhaps plausible interactions are predicted between domains PF05930 and PF13356 of prophage protein AlpA and several phage integrase proteins as well as between domain PF13518 with PF13817, the former a HTH domain commonly associated with transposase domains and the latter a transposase domain.

Insufficient information on the function of two domain pairs and their associated proteins does not allow us to draw any conclusions on the plausibility of interaction. These are for domains PF02021 and PF13335 of proteins YraN and YifB and domains PF01906 with PF02796, the former a metal binding domain and the latter a domain found in site specific recombinases.

Lastly, we find three proposed interactions between domains found in ribosomal proteins RL36, RL34 and RL32 (PF00444, PF00468, PF01783) and also a protein of unknown function YidD (PF01809). We consider these to be likely false positive predictions since we previously observed spurious results for members of very large macromolecular complexes such as the ribosome [10]. At least the interaction between YidD and RL36 seems plausible, as the former has been suggested to play a role as membrane protein insertion factor [55].

In summary, we are able to recapitulate several known or plausible but structurally unresolved interactions and find several examples of interaction that should be of interest for future experimental studies.

## Discussion

In this work, we propose a coevolutionary analysis connecting signals at the phylogenetic level (correlated presence of domain pairs across genomes) with the residue level (correlated occurrence of amino acids between proteins). At the phylogenetic level, we introduce the concept of *phyletic couplings*: by using a global statistical model, we are able to disentangle direct and indirect correlations in the presence and absence of protein domains across more than 1000 fully sequenced representative bacterial species. Couplings substantially increase the capacity to find relations between domains beyond correlations; these relations can be physical interactions, but also genomic co-localization (and thus likely functional relations). Standard correlation measures used in phylogenetic profiling only reach 30–50% of true positives between the first 1000 predictions. In contrast the positive predictive value of phylogenetic couplings reaches about 80%. The results are very robust: when applying the same methodology to all 9358 Pfam domains appearing in the bacteria, and selecting only later the couplings between domains present in *E*. *coli*, couplings have 96% correlation with the couplings found by the procedure described before.

The high accuracy of phyletic couplings in predicting domain-domain relations, along with the robustness of these couplings when extensively changing the data set, allows us to hypothesize that large couplings not corresponding to known relations predict novel, unknown relations. A list of the 100 first predictions is provided in Tables A and B in *Supplement* S1 Text.

As mentioned, a large phyletic coupling does not automatically imply a direct physical interaction. Two proteins may have a strong phyletic coupling because they belong to the same multi-protein complex, without touching each other. They may have a strong phyletic coupling, because they act within the same biological process or pathway, again without any direct interaction. To refine the results and predict physical interactions, we have added a coevolutionary analysis on the scale of residue-residue covariation, as provided by DCA, in the version with paralog matching as recently proposed in [27]. We find that 72% of the 500 phylogenetically most coupled pairs correspond to large enough alignments to run DCA, and 12.5% of these have significant DCA scores.

These domain pairs are our strongest candidates for predicted domain-domain interactions. Since they are not co-localized in the same protein, they also provide predictions for new protein-protein interactions. In a detailed discussion, we have shown that most of the 24 pairs with a DCA score larger than 0.2, which result from the first 200 PhyDCA predictions have a sensible biological interpretation and, in principle, could be tested experimentally.

Similarly, negative phylogenetic couplings appear to be biologically reasonable. They disfavor the joint presence of two domains within the same genome. In our analysis of the pairs of the strongest negative couplings, presented above in *Results*, we actually find many pairs having the same functionality, including documented pairs of convergent evolution. Some pairs actually are of unknown function, and our method might help to transfer functional annotations from one domain to the other.

**Fig 5:**
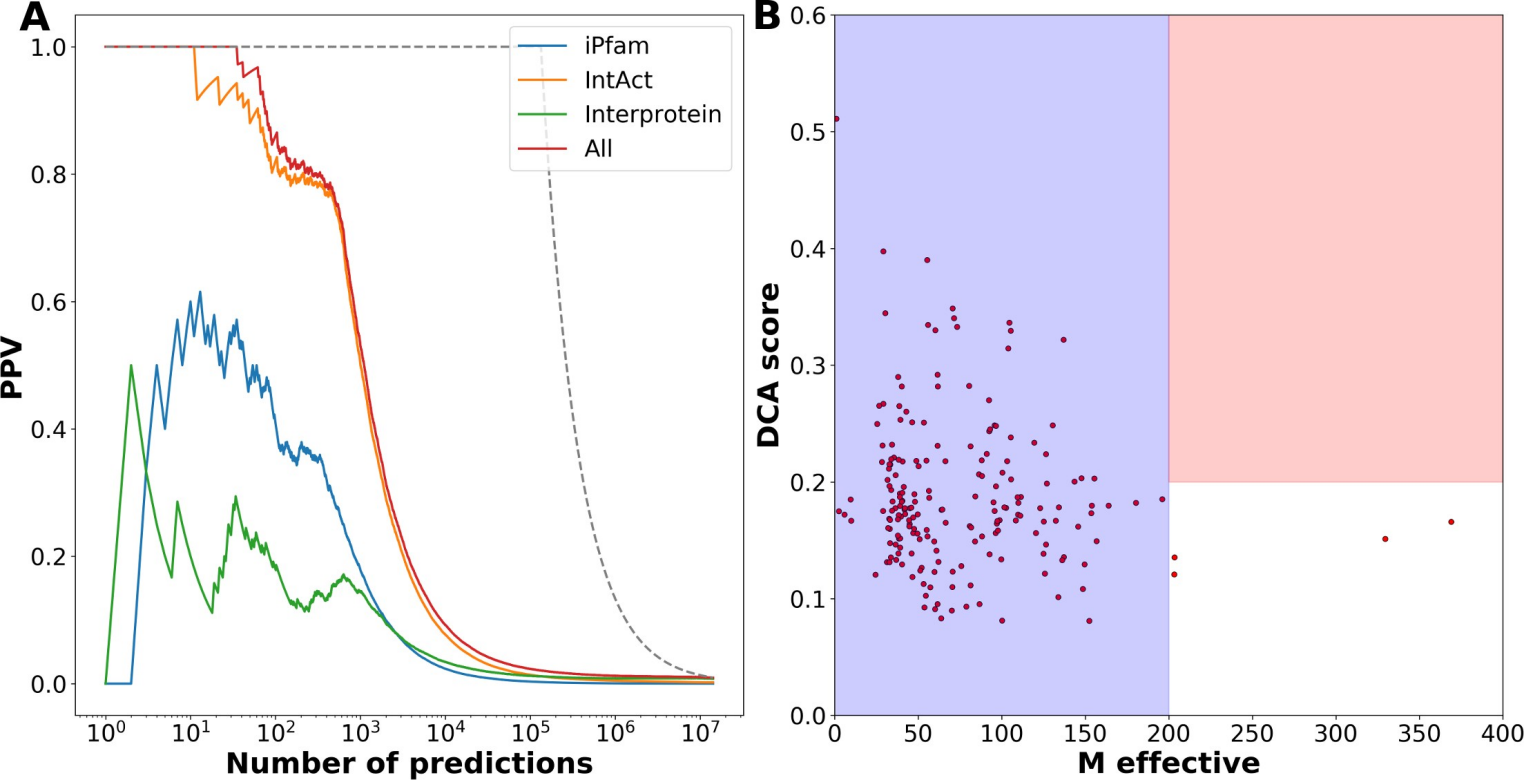
Performance of our multi-scale coevolutionary analysis for human protein domains. **–**Panel A shows the positive predictive value of the phyletic couplings for predicting positive domain-domain relationships (including protein architecture, iPfam and human IntAct entries). While there is a clear overrepresentation of intra-protein localization in between the highest-scoring domain pairs, also physical interactions as captured by iPfam and IntAct are enriched in particular in the first ca. 10^3^ phyletic couplings. The overall performance is coherent with the one found in the bacteria. Panel B shows the paralog-matching and DCA results for the 200 most coupled domain pairs, which are not in the positive-relation dataset. We observe that currently the joint MSA are too small (Meff < 200) to allow for a reliable application of DCA to detect inter-protein residue-scale coevolution.

An important extension would be the application of our approach beyond the bacteria. Bacteria, due to their compact genomes, are overrepresented in genomic databases, including the Pfam database, which we used for our analysis. To test the applicability to higher organisms, we have repeated the same procedure, concentrating on eukaryotic genomes and taking humans as the reference species. Data get much less abundant; the phylogenetic profile matrix now contains 5343 domains as compared to only 481 eukaryotic species. Still, phyletic couplings, when compared to a positive list extracted from domain architectures of human proteins (co-localization in one protein), from iPfam [44] and human entries in IntAct [56], show a similar performance as the bacterial case, cf. Fig 5A: 75% of the first 1000 couplings correspond to known domain-domain relations. Entries corresponding to protein-protein interactions (iPfam, IntAct) are again significantly enriched, even if to a lesser extent than in the bacterial case. The most important difference emerges, however, when using paralog matching and DCA on the 200 most coupled predictions (i.e. pairs with strong phylogenetic coupling but not belonging to the positive list), cf. Fig 5B: Only 2–4 have sequence numbers that allow for reliable DCA results. More eukaryotic genomes are urgently needed to carry out our full procedure also in higher species.

To conclude, our work illustrates the potential of combining rapidly growing genomic databases and statistical modeling: the increasing number of fully sequenced genomes allows for extracting rich *samples for the variability in protein content and protein seque*nces across hundreds and thousands of species; their statistical analysis helps us to detect multiple scales of coevolution between interacting or functionally related proteins.

The *genomic scale* explores the correlated presence or absence of proteins (in the sense of homologous protein families) across species. This correlation has been used before within phylogenetic profiling to detect functional relations or direct interactions between proteins. Within our work, we propose to infer direct phyletic couplings via global statistical models, and prove that this concept strongly improves our capacity to detect protein relations over local correlation measures.

However, phylogenetic couplings cannot distinguish between functional relations or direct interactions between proteins. This problem can–at least partially–be resolved at the *residue scale* of inter-protein coevolution. Interacting proteins show a correlated usage of amino acids across their interface, and again global statistical modeling approaches like DCA have been used to discriminate between interacting and non-interacting protein pairs.

Since the computational cost of the residue-scale analysis is high, it is possible to analyze all pairings between 10–50 proteins, but not all pairs between thousands of proteins forming a species’ proteome. It is the combination of both scales, which allows us to first explore the genomic scale and then refine promising results at the residue scale. Doing so, we have provided a number of biologically sensible predictions for currently unknown protein-protein interactions. We provide a list of these predictions, which in turn may be tested directly.

Last but not least, we want to mention that the analysis of both scales of coevolution is done independently, i.e., in a modular way, even if using a common mathematical-statistical framework. In principle it is therefore possible to improve each single component on its own. We might, e.g., come up with a phylogenetically better-founded version of PhyDCA (i.e. combining the spirit of CoPAP and PhyDCA), to generate better candidates for novel domain-domain interactions. Similarly, improvement in paralog matching and DCA-based interaction prediction might lead to a more sensitive treatment of these candidates.

## Methods

### Phylogenetic profiles

Data are extracted from the Pfam 30.0 database [31]. For each of the 1,041 bacterial genomes present in Pfam, we extract all appearing protein-domain families, accounting to a total of 9,358 Pfam families. A restriction to *Escherichia coli* as reference genome (i.e. counting only domains contained in *E*. *coli*) reduces this to 2682 domain families. Since we are interested in the *correlated* presence / absence of domains across species, we remove all domain families with less than 5% or more than 95%, keeping only domains with at least 53 and at most 988 appearances. This removes in particular omnipresent domains related, e.g., to replication, transcription and translation. The final phylogenetic profile matrix (PPM) is a binary matrix containing *M* = 1,041 bacteria and *N* = 2,041 domains. Entries are one if a domain is present in a species (at least once), and zero if it is absent. Note that a zero entry typically indicates a true absence of the domain in a genome, since the profile matrix is entirely built on fully sequenced genomes.

In standard phylogenetic profiling [5], correlations between domains are evaluated via the Hamming distance, Pearson correlation or p-values of Fisher’s exact test, cf. the *Supplement* S1 Text for the definitions in the context of our work.

### Phyletic couplings

In analogy to the direct-coupling analysis on the level of amino-acid sequences, we model the phylogenetic profiles via the maximum-entropy principle by a global statistical model

$$P(n_{1},\ldots ,n_{N})=\frac{1}{Z}\exp\left\{\sum_{i<j}J_{ij}n_{i}n_{j}+\sum_{i}h_{i}n_{i}\right\}$$

with (*n*_*1*_, …,*n*_*N*_) being a binary vector characterizing the presence (*n*_*i*_ = 1) or absence (*n*_*i*_ = 0) of domain *i* in a species, and *Z* is a normalization constant also known as partition function in statistical physics. The *phyletic couplings J*_*ij*_ and *biases h*_*i*_ are to be determined such that the model *P* reproduces the one- and two-column statistics of the PPM (*n*_*i*_^*a*^)_i = 1,…,N; a = 1,…,M_:

$$f_{i}=\frac{1}{M}\sum_{a=1}^{M}n_{i}^{a}$$

$$f_{ij}=\frac{1}{M}\sum_{a=1}^{M}n_{i}^{a}n_{j}^{a}$$

with *f*_*i*_ being the fraction of genomes in the PPM carrying domain *i*, and *f*_*ij*_ the fraction of genomes containing both domains *i* and *j* simultaneously. While the exact determination of the marginal distributions of P requires exponential-time computations, we apply the mean-field (MF) and pseudo-likelihood maximization (PLM) approximations successfully used in the context of DCA [19, 57]; cf. the *Supplement* S1 Text for technical details. Due to the high dimensionality of the problems (*N* = 2041−9358), more precise methods based on Boltzmann machine learning, cf. [32], become computationally prohibitive. Strong positive couplings favor the joint presence or joint absence of two domains, signaling therefore a positive association between the two (genomic colocalization, functional relation, domain-domain interaction). Strong negative couplings favor the appearance of only one out of the two domains, signaling domains of similar function (e.g. convergent evolution). Before analyzing the phyletic couplings, we apply the so-called Average Product Correction (APC) [58], cf. *Supplement* S1 Text. APC is widely used to suppress spurious couplings resulting from the heterogeneous conservation statistics domain families across genomes (cf. [59]) as compared to functional couplings. In the case of PhyDCA, it has a limited effect, as is shown in Fig A of *Supplement* S1 Text.

### Direct coupling analysis of inter-protein residue coevolution

To assess the coevolution on the finer scale of residue-residue coevolution, we have applied exactly the progressive matching and analysis procedure recently published by part of us in [27], details about the procedure are given in the *Supplement* S1 Text. It starts with two domain alignments, containing only bacterial protein sequences. It matches sequences between the domain families, such that (a) only sequences from the same species are matched and (b) the total inter-family covariation signal is maximized. Results are considered positive if (i) the effective number of matched sequences (at 80% seq ID) exceeds 200 and (ii) the covariation score exceeds 0.2. It has been established in [10, 27] that larger scores are rarely obtained by unrelated protein families. Note that a smaller score may be related to a functional relationship rather than a physical protein-protein interaction, or also to a small or non-conserved interaction interface [43].

### Known domain-domain relationships

To assess the accuracy of our predictions, we have compiled a number of known relationships (provided in *Supplement* S1 Text). They come from different databases, the same domain-domain pair may appear multiple times, but it is counted only once in the final list of positives:

1. *Intra-protein localization*: From the Pfam database [31], we have extracted a list of domain pairs, which co-occur inside single proteins in *E*. *coli*. Out of 3,116 proteins, 952 contained multiple domains, giving rise to 799 distinct domain-domain relations.
2. *Intra-operon localization*: Proteins, which are co-localized inside operons, frequently share at least part of their biological function. Using a list of operons from *E*. *coli* [60], we compiled a list of 4,087 colocalized domain pairs.
3. *Protein-protein interaction*: The IntAct database [56] contains 5,318 pairs of experimentally found protein-protein interactions. At the domain level, we pair all domains in one protein with all domains in the second protein (adding possibly unrelated domain pairs to those interacting), obtaining 3,070 domain pairs.
4. *Domain-domain contacts in 3D structures*: The iPfam database [44] contains domain-domain interactions extracted from structural domain-domain contacts in experimentally determined complex structures in the PDB. We included intra- and inter-chain contacts, i.e. domain-domain contacts inside a protein or between two proteins. Note that this list does not refer to *E*. *coli* as reference genome. In total, this accounts to 545 know relationships.
5. *Metabolic relationships between enzymes*: Using the reconstruction iJR904 of *E*. *coli’s* metabolic network [61] and filtering out “currency” metabolites involved in more than 50 reactions (such as water, ATP etc.), we considered three relationships:
  - *common substrate–* pairs of enzymes catalyzing reactions with at least one common substrate;
  - *common product–*pairs of enzymes catalyzing reactions with at least one common product;
  - *reaction chains–*pairs of enzymes catalyzing subsequent reactions, i.e., one product of one reaction is substrate of the second.

This lead to a total of 677 known relationships.

The total list contains 8,091 domain-domain pairs, as compared to the 2,081,820 possible pairs, which can be formed out of the 2,041 domains in our PPM.

## Supporting information

S1 Text Supplementary information. This text contains technical details about the data, the computational analysis tools, and supporting results and figures.
(PDF)

## References

[1] Braun P, Tasan M, Dreze M, Barrios-Rodiles M, Lemmens I, Yu H, et al An experimentally derived confidence score for binary protein-protein interactions. Nat Methods. 2009;6(1):91–7. 10.1038/nmeth.1281 19060903 PMC2976677

[2] Harrington ED, Jensen LJ, Bork P. Predicting biological networks from genomic data. FEBS Lett. 2008;582(8):1251–8. 10.1016/j.febslet.2008.02.033 18294967

[3] Valencia A, Pazos F. Computational methods for the prediction of protein interactions. Curr Opin Struct Biol. 2002;12(3):368–73. 12127457 10.1016/s0959-440x(02)00333-0

[4] Pellegrini M, Marcotte EM, Thompson MJ, Eisenberg D, Yeates TO. Assigning protein functions by comparative genome analysis: protein phylogenetic profiles. Proc Natl Acad Sci U S A. 1999;96(8):4285–8. 10.1073/pnas.96.8.4285 10200254 PMC16324

[5] Pellegrini M. Using phylogenetic profiles to predict functional relationships. Methods Mol Biol. 2012;804:167–77. 10.1007/978-1-61779-361-5_9 22144153

[6] Juan D, Pazos F, Valencia A. High-confidence prediction of global interactomes based on genome-wide coevolutionary networks. Proc Natl Acad Sci U S A. 2008;105(3):934–9. 10.1073/pnas.0709671105 18199838 PMC2242690

[7] Pazos F, Valencia A. In silico two-hybrid system for the selection of physically interacting protein pairs. Proteins. 2002;47(2):219–27. 11933068 10.1002/prot.10074

[8] Burger L, van Nimwegen E. Accurate prediction of protein-protein interactions from sequence alignments using a Bayesian method. Mol Syst Biol. 2008;4:165 10.1038/msb4100203 18277381 PMC2267735

[9] Procaccini A, Lunt B, Szurmant H, Hwa T, Weigt M. Dissecting the specificity of protein-protein interaction in bacterial two-component signaling: orphans and crosstalks. PLoS One. 2011;6(5):e19729 10.1371/journal.pone.0019729 21573011 PMC3090404

[10] Feinauer C, Szurmant H, Weigt M, Pagnani A. Inter-Protein Sequence Co-Evolution Predicts Known Physical Interactions in Bacterial Ribosomes and the Trp Operon. PLoS One. 2016;11(2):e0149166 10.1371/journal.pone.0149166 26882169 PMC4755613

[11] Spencer M, Sangaralingam A. A phylogenetic mixture model for gene family loss in parasitic bacteria. Mol Biol Evol. 2009;26(8):1901–8. 10.1093/molbev/msp102 19435739

[12] Cohen O, Pupko T. Inference of gain and loss events from phyletic patterns using stochastic mapping and maximum parsimony—a simulation study. Genome Biol Evol. 2011;3:1265–75. 10.1093/gbe/evr101 21971516 PMC3215202

[13] Cohen O, Ashkenazy H, Burstein D, Pupko T. Uncovering the co-evolutionary network among prokaryotic genes. Bioinformatics. 2012;28(18):i389–i94. 10.1093/bioinformatics/bts396 22962457 PMC3436823

[14] Kim PJ, Price ND. Genetic co-occurrence network across sequenced microbes. PLoS Comput Biol. 2011;7(12):e1002340 10.1371/journal.pcbi.1002340 22219725 PMC3248385

[15] Rivoire O. Elements of coevolution in biological sequences. Phys Rev Lett. 2013;110(17):178102 10.1103/PhysRevLett.110.178102 23679784

[16] de Juan D, Pazos F, Valencia A. Emerging methods in protein co-evolution. Nat Rev Genet. 2013;14(4):249–61. 10.1038/nrg3414 23458856

[17] Szurmant H, Weigt M. Inter-residue, inter-protein and inter-family coevolution: bridging the scales. Curr Opin Struct Biol. 2017;50:26–32. 10.1016/j.sbi.2017.10.014 29101847 PMC5940578

[18] Weigt M, White RA, Szurmant H, Hoch JA, Hwa T. Identification of direct residue contacts in protein-protein interaction by message passing. Proc Natl Acad Sci U S A. 2009;106(1):67–72. 10.1073/pnas.0805923106 19116270 PMC2629192

[19] Morcos F, Pagnani A, Lunt B, Bertolino A, Marks DS, Sander C, et al Direct-coupling analysis of residue coevolution captures native contacts across many protein families. Proc Natl Acad Sci U S A. 2011;108(49):E1293–301. 10.1073/pnas.1111471108 22106262 PMC3241805

[20] Kamisetty H, Ovchinnikov S, Baker D. Assessing the utility of coevolution-based residue-residue contact predictions in a sequence- and structure-rich era. Proc Natl Acad Sci U S A. 2013;110(39):15674–9. 10.1073/pnas.1314045110 24009338 PMC3785744

[21] Jones DT, Buchan DWA, Cozzetto D, Pontil M. PSICOV: precise structural contact prediction using sparse inverse covariance estimation on large multiple sequence alignments. Bioinformatics. 2012;28(2):184–90. 10.1093/bioinformatics/btr638 22101153

[22] Schug A, Weigt M, Onuchic JN, Hwa T, Szurmant H. High-resolution protein complexes from integrating genomic information with molecular simulation. Proc Natl Acad Sci U S A. 2009;106(52):22124–9. 10.1073/pnas.0912100106 20018738 PMC2799721

[23] Hopf TA, Scharfe CPI, Rodrigues JPGLM, Green AG, Kohlbacher O, Sander C, et al Sequence co-evolution gives 3D contacts and structures of protein complexes. Elife. 2014;3. 10.7554/eLife.03430 PMC4360534 25255213

[24] Ovchinnikov S, Kamisetty H, Baker D. Robust and accurate prediction of residue-residue interactions across protein interfaces using evolutionary information. Elife. 2014;3:e02030 10.7554/eLife.02030 24842992 PMC4034769

[25] Rodriguez-Rivas J, Marsili S, Juan D, Valencia A. Conservation of coevolving protein interfaces bridges prokaryote-eukaryote homologies in the twilight zone. Proc Natl Acad Sci U S A. 2016;113:15018–23. 10.1073/pnas.1611861114 27965389 PMC5206536

[26] Bitbol A-F, Dwyer RS, Colwell LJ, Wingreen NS. Inferring interaction partners from protein sequences. Proc Natl Acad Sci U S A. 2016;113(43):12180–5. 10.1073/pnas.1606762113 27663738 PMC5087060

[27] Gueudre T, Baldassi C, Zamparo M, Weigt M, Pagnani A. Simultaneous identification of specifically interacting paralogs and interprotein contacts by direct coupling analysis. Proc Natl Acad Sci U S A. 2016;113(43):12186–91. 10.1073/pnas.1607570113 27729520 PMC5087065

[28] Yeang CH. Identifying coevolving partners from paralogous gene families. Evol Bioinform Online. 2008;4:97–107. 19204811 10.4137/ebo.s621 PMC2614191

[29] Cohen O, Ashkenazy H, Levy Karin E, Burstein D, Pupko T. CoPAP: Coevolution of presence-absence patterns. Nucleic Acids Res. 2013;41(Web Server issue):W232–7. 10.1093/nar/gkt471 23748951 PMC3692100

[30] Pagel P, Wong P, Frishman D. A domain interaction map based on phylogenetic profiling. J Mol Biol. 2004;344(5):1331–46. 10.1016/j.jmb.2004.10.019 15561146

[31] Finn RD, Coggill P, Eberhardt RY, Eddy SR, Mistry J, Mitchell AL, et al The Pfam protein families database: towards a more sustainable future. Nucleic Acids Res. 2016;44(D1):D279–85. 10.1093/nar/gkv1344 26673716 PMC4702930

[32] Cocco S, Feinauer C, Figliuzzi M, Monasson R, Weigt M. Inverse Statistical Physics of Protein Sequences: A Key Issues Review. arXiv preprint arXiv:1703:01222 2017. 10.1088/1361-6633/aa9965 29120346

[33] Qin C, Colwell LJ. Power law tails in phylogenetic systems. Proc Natl Acad Sci U S A. 2018;115(4):690–5. 10.1073/pnas.1711913115 29311320 PMC5789915

[34] Cocco S, Monasson R, Weigt M. From principal component to direct coupling analysis of coevolution in proteins: low-eigenvalue modes are needed for structure prediction. PLoS Comput Biol. 2013;9(8):e1003176 10.1371/journal.pcbi.1003176 23990764 PMC3749948

[35] Morett E, Korbel JO, Rajan E, Saab-Rincon G, Olvera L, Olvera M, et al Systematic discovery of analogous enzymes in thiamin biosynthesis. Nat Biotechnol. 2003;21(7):790–5. 10.1038/nbt834 12794638

[36] Myllykallio H, Lipowski G, Leduc D, Filee J, Forterre P, Liebl U. An alternative flavin-dependent mechanism for thymidylate synthesis. Science. 2002;297(5578):105–7. 10.1126/science.1072113 12029065

[37] Herrmann KM. The shikimate pathway as an entry to aromatic secondary metabolism. Plant Physiol. 1995;107(1):7–12. 10.1104/pp.107.1.7 7870841 PMC161158

[38] Sebastian M, Ammerman JW. The alkaline phosphatase PhoX is more widely distributed in marine bacteria than the classical PhoA. ISME J. 2009;3(5):563–72. 10.1038/ismej.2009.10 19212430

[39] Okamura K, Hagiwara-Takeuchi Y, Li T, Vu TH, Hirai M, Hattori M, et al Comparative genome analysis of the mouse imprinted gene impact and its nonimprinted human homolog IMPACT: toward the structural basis for species-specific imprinting. Genome Res. 2000;10(12):1878–89. 10.1101/gr.139200 11116084

[40] Demaegd D, Colinet AS, Deschamps A, Morsomme P. Molecular evolution of a novel family of putative calcium transporters. PLoS One. 2014;9(6):e100851 10.1371/journal.pone.0100851 24955841 PMC4067407

[41] Waters LS, Sandoval M, Storz G. The Escherichia coli MntR miniregulon includes genes encoding a small protein and an efflux pump required for manganese homeostasis. J Bacteriol. 2011;193(21):5887–97. 10.1128/JB.05872-11 21908668 PMC3194919

[42] Shi HJ, Wu AZ, Santos M, Feng ZM, Huang L, Chen YM, et al Cloning and characterization of rat spermatid protein SSP411: a thioredoxin-like protein. J Androl. 2004;25(4):479–93. 15223837 10.1002/j.1939-4640.2004.tb02819.x

[43] Uguzzoni G, John Lovis S, Oteri F, Schug A, Szurmant H, Weigt M. Large-scale identification of coevolution signals across homo-oligomeric protein interfaces by direct coupling analysis. Proc Natl Acad Sci U S A. 2017;114(13):E2662–E71. 10.1073/pnas.1615068114 28289198 PMC5380090

[44] Finn RD, Miller BL, Clements J, Bateman A. iPfam: a database of protein family and domain interactions found in the Protein Data Bank. Nucleic Acids Res. 2014;42(Database issue):D364–73. 10.1093/nar/gkt1210 24297255 PMC3965099

[45] Greie JC. The KdpFABC complex from Escherichia coli: a chimeric K+ transporter merging ion pumps with ion channels. Eur J Cell Biol. 2011;90(9):705–10. 10.1016/j.ejcb.2011.04.011 21684627

[46] Siegel AR, Wemmer DE. Role of the sigma54 Activator Interacting Domain in Bacterial Transcription Initiation. J Mol Biol. 2016;428(23):4669–85. 10.1016/j.jmb.2016.10.007 27732872 PMC5116005

[47] Vales LD, Rabin BA, Chase JW. Subunit structure of Escherichia coli exonuclease VII. J Biol Chem. 1982;257(15):8799–805. 6284744

[48] Abendroth J, Murphy P, Sandkvist M, Bagdasarian M, Hol WG. The X-ray structure of the type II secretion system complex formed by the N-terminal domain of EpsE and the cytoplasmic domain of EpsL of Vibrio cholerae. J Mol Biol. 2005;348(4):845–55. 10.1016/j.jmb.2005.02.061 15843017

[49] Strom MS, Nunn DN, Lory S. Posttranslational processing of type IV prepilin and homologs by PilD of Pseudomonas aeruginosa. Methods Enzymol. 1994;235:527–40. 10.1016/0076-6879(94)35168-6 8057924

[50] Sakai D, Komano T. The pilL and pilN genes of IncI1 plasmids R64 and ColIb-P9 encode outer membrane lipoproteins responsible for thin pilus biogenesis. Plasmid. 2000;43(2):149–52. 10.1006/plas.1999.1434 10686134

[51] Labahn J, Scharer OD, Long A, Ezaz-Nikpay K, Verdine GL, Ellenberger TE. Structural basis for the excision repair of alkylation-damaged DNA. Cell. 1996;86(2):321–9. 10.1016/s0092-8674(00)80103-8 8706136

[52] Mielecki D, Grzesiuk E. Ada response—a strategy for repair of alkylated DNA in bacteria. FEMS Microbiol Lett. 2014;355(1):1–11. 10.1111/1574-6968.12462 24810496 PMC4437013

[53] Zhang Y, Inouye M. The inhibitory mechanism of protein synthesis by YoeB, an Escherichia coli toxin. J Biol Chem. 2009;284(11):6627–38. 10.1074/jbc.M808779200 19124462 PMC2652283

[54] Jorgensen MG, Pandey DP, Jaskolska M, Gerdes K. HicA of Escherichia coli defines a novel family of translation-independent mRNA interferases in bacteria and archaea. J Bacteriol. 2009;191(4):1191–9. 10.1128/JB.01013-08 19060138 PMC2631989

[55] Yu Z, Laven M, Klepsch M, de Gier JW, Bitter W, van Ulsen P, et al Role for Escherichia coli YidD in membrane protein insertion. J Bacteriol. 2011;193(19):5242–51. 10.1128/JB.05429-11 21803992 PMC3187451

[56] Kerrien S, Aranda B, Breuza L, Bridge A, Broackes-Carter F, Chen C, et al The IntAct molecular interaction database in 2012. Nucleic Acids Res. 2012;40(Database issue):D841–6. 10.1093/nar/gkr1088 22121220 PMC3245075

[57] Ekeberg M, Lovkvist C, Lan Y, Weigt M, Aurell E. Improved contact prediction in proteins: using pseudolikelihoods to infer Potts models. Phys Rev E Stat Nonlin Soft Matter Phys. 2013;87(1):012707 10.1103/PhysRevE.87.012707 23410359

[58] Dunn SD, Wahl LM, Gloor GB. Mutual information without the influence of phylogeny or entropy dramatically improves residue contact prediction. Bioinformatics. 2008;24(3):333–40. 10.1093/bioinformatics/btm604 18057019

[59] Talavera D, Lovell SC, Whelan S. Covariation Is a Poor Measure of Molecular Coevolution. Mol Biol Evol. 2015;32(9):2456–68. 10.1093/molbev/msv109 25944916 PMC4540965

[60] Taboada B, Ciria R, Martinez-Guerrero CE, Merino E. ProOpDB: Prokaryotic Operon DataBase. Nucleic Acids Res. 2012;40(Database issue):D627–31. 10.1093/nar/gkr1020 22096236 PMC3245079

[61] Reed JL, Vo TD, Schilling CH, Palsson BO. An expanded genome-scale model of Escherichia coli K-12 (iJR904 GSM/GPR). Genome Biol. 2003;4(9):R54 10.1186/gb-2003-4-9-r54 12952533 PMC193654

