# Supplementary material for: A multi-scale coevolutionary approach to predict interactions between protein domains
Source: PLoS Comput Biol. 2019 Oct 21;15(10):e1006891. doi: 10.1371/journal.pcbi.1006891 (PMC6822775; doi:10.1371/journal.pcbi.1006891)
Supplement: S1 Text — This text contains technical details about the data, the computational analysis tools, and supporting results and figures. (PDF) [file pcbi.1006891.s001.pdf]

SUPPLEMENTARY INFORMATION

A multi-scale coevolutionary approach to predict protein-protein interactions

Giancarlo Croce[1], Thomas Gueudré[2], Maria Virginia Ruiz Cuevas[1], Victoria Keidel[3], Matteo Figliuzzi[1], Hendrik Szurmant[3], Martin Weigt[1]

[1] Sorbonne Université, CNRS, Institut de Biologie Paris Seine, Biologie Computationnelle et Quantitative - LCQB, 75005 Paris, France

[2] Italian Institute for Genomic Medicine, 10126 Torino, Italy

[3] Department of Basic Medical Sciences, College of Osteopathic Medicine of the Pacific, Western University of Health Sciences, Pomona CA 91766, USA

Contents

A Input data **1**

B Similarity measures **2**

C Inference techniques **3**
- C.1 Pre-processing the data 4
- C.2 Mean Field (MF) 4
- C.3 Pseudolikehood (PLM) 4
- C.4 Average product correction (APC) 5

D Results **6**

E Progressive paralog matching procedure **8**
- E.1 Network analysis 14

F All bacteria **17**
- F.1 Negative phyletic couplings 19

G Eukaryotic genomes: human as reference species **19**

A Input data

The starting point of our analysis is the phylogenetic profile matrix (PPM): a binary matrix $(n_i^a)_{i=1,\dots,N}^{a=1,\dots,M}$ whose entries capture the presence ($n_i^a = 1$) or absence ($n_i^a = 0$) of a domain i in genome a, with $a = 1, \dots, M$ (M being the number of genomes) and $i = 1, \dots, N$ (N being the number of domains). As discussed in the main text, the domains (the columns of the PPM) are then

compared with each other to look for functionally related domains. The data we use are extracted from the Pfam 30.0 database (version of July 2016) [2], and assigned to bacterial or eukaryotic species using the Uniprot species list available on (`http://www.uniprot.org/docs/speclist`).

B Similarity measures

In standard *phylogenetic profiling* [5] the correlations between the columns $(\mathbf{n}_i, \mathbf{n}_j)$ describing a pair of domains are usually evaluated via the Hamming distance, Pearson correlation or the p-value of the Fisher's exact test. We briefly describe each below.

- **Hamming distance**: counts the number of bits which differ between two binary strings $\underline{n}_i, \underline{n}_j$ divided by the total number of domains, i.e. the number of species containing exactly one of the two domains,

$$d_H(\underline{n}_i, \underline{n}_j) = |\{n_i^a \neq n_j^a, \quad a = 1, \ldots, M\}|/M \tag{1}$$

- **Pearson Correlation**: measures the linear dependence between two domains $\underline{n}_i, \underline{n}_j$. It is defined as:

$$r(\underline{n}_i, \underline{n}_j) = \frac{\sum_{a=1}^{M}(n_i^a - \bar{n_i})(n_j^b - \bar{n_j})}{\sqrt{\sum_{a=1}^{M}(n_i^a - \bar{n_i})^2}\sqrt{\sum_{a=1}^{M}(n_j^a - \bar{n_j})^2}} \tag{2}$$

- **p-value of Fisher Test**: for each couple $(\underline{n}_i, \underline{n}_j)$ we construct an auxiliary 2×2 matrix

$$\begin{pmatrix} M_{1,1} & M_{1,2} \\ M_{2,1} & M_{2,2} \end{pmatrix} \tag{3}$$

  with:

  $M_{1,1}$: Number of species that do not have neither domain i nor j

  $M_{1,2}$: Number of species that have the i but not j

  $M_{2,1}$: Number of species that do not have i but have j

  $M_{2,2}$: Number of species that have both i and j.

  We define $R_i = \sum_j M_{i,j}$, $C_j = \sum_i M_{i,j}$, and we have $M = \sum_{i,j} M_{i,j}$. We calculate the conditional probability of getting the actual matrix given the particular row and column sums:

$$P_{\text{cutoff}} = \frac{\binom{R_1}{M_{11}}\binom{R_2}{M_{21}}}{\binom{M}{C_1}} = \frac{R_1!R_2!C_1!C_2!}{M!M_{1,1}!M_{1,2}!M_{2,1}!M_{2,2}!} \tag{4}$$

  which is a multivariate generalization of the hyper-geometric distribution. Theoretically, we analyse all the matrices of non negative integers consistent with the marginals R_i, C_j and M, and for each of them we calculate

the p-value Eq.(4). The p-value of the test is the sum of all p-values which are $P \leq P_{cutoff}$. Small p-values thus indicate atypical cases related to correlations between the distributions of the two domains across species.

C Inference techniques

As a simple but meaningful statistical model we consider a pairwise lattice-gas Ising model with the following Hamiltonian:

$$H(\mathbf{n}) = -\sum_{i=1}^{N} h_i n_i - \sum_{i<j}^{N} J_{ij} n_i n_j \ . \tag{5}$$

It shall model the statistical variability of the phylogenetic profile $\mathbf{n} = (n_1, ..., n_N)$ of an entire species. The binary variables n_i ($i = 1, \ldots, N$, with N being the total number of domains) can have values $n_i = 1$ i.e. the domain is present, or $n_i = 0$ otherwise. Each row of the PPM has this form, and H can thus be evaluated for each species contained in the PPM.

To infer the Ising model Eq.(5) reproducing the empirical one- and two-column statistics, we assume as working hypothesis that the phylogenetic profile matrix PPM is composed by configuration sampled from the equilibrium Boltzmann-Gibbs distribution (in the inference process we are free to set the inverse temperature $\beta = 1$):

$$P(\mathbf{n}) = \frac{1}{Z} \exp[-H(\mathbf{n})]. \tag{6}$$

In the inference procedure we look for parameters $\{\mathbf{h}, \mathbf{J}\}$ (respectively the *fields* and the *phyletic couplings*) that maximize the log-likelihood:

$$L(\mathbf{h}, \mathbf{J}) = \frac{1}{M} \sum_{m}^{M} w_m \log[P(\mathbf{n}|\mathbf{h}, \mathbf{J})] \tag{7}$$

where w_m are reweighting coefficients defined below in Section C.1. Nevertheless the exact maximization procedure requires the determination of the marginals of $P(\mathbf{n})$ for single variables and pairs, which is highly computationally expensive. In the last years [9] a variety of efficient and accurate approximation methods have been proposed: in this work we use the Mean Field (MF) and pseudo-likelihood maximization (PLM) approximations, which, as showed in Figure 2 of the main text, give similar results on our datasets. We briefly describe each one below.

We also mention that we developed an implementation of Phyletic-Coupling Analysis *PhyDCA*, in Julia, a new open-source language [15]. The package can be downloaded at `https://github.com/GiancarloCroce/PhyCA`.

C.1 Pre-processing the data

In order to reduce sampling biases, a simple correction is to associate to each phylogenetic profile of a species $\mathbf{n}^a$ the weight $w_a = 1/m_a$ with m_a the number of similar profiles (including a itself):

$$m_a = |\{\mathbf{n}^b | 1 \leq b \leq M, d_H(\mathbf{n}^a, \mathbf{n}^b) < \theta)\}| \tag{8}$$

with $d_H(\mathbf{n}^a, \mathbf{n}^b)$ being the Hamming distance and θ a similarity threshold. It has been empirically observed that setting $\theta = 0.8$ slightly improves the PPV. The sum of all the weights $M_{eff} = \sum_{a=1}^{M} w_a$, represents the effective number of phylogenetic profiles counted as independent in the analysis.

C.2 Mean Field (MF)

The Mean Field approximation is currently the fastest approximate inference scheme even if not accurate when the number of samples is small nor when the interactions are strong [9]. The phyletic couplings J_{ij} are inferred from the empirical correlation matrix: $J_{ij} = -(C^{-1})_{ij}$ for $i \neq j$, with

$$C_{ij} = (f_{ij} - f_i f_j) \tag{9}$$

$$f_i = \frac{\lambda}{2} + (1-\lambda)\left(\frac{1}{Meff}\sum_{a=1}^{M} w_a n_i^a\right) \tag{10}$$

$$f_{ij} = \frac{\lambda}{4} + (1-\lambda)\left(\frac{1}{Meff}\sum_{a=1}^{M} w_a n_i^a n_j^a\right) \tag{11}$$

where λ is a pseudocount that we set to $\lambda = \frac{1}{4}$ which we empirically found to produce the optimal PPV.

C.3 Pseudolikehood (PLM)

Adapted in [4] to protein sequences, the approximation consists in replacing the Boltzmann-Gibbs measure with the following conditional probability distribution:

$$P(n_i|\mathbf{n}_{\backslash i}) = \frac{\exp\left\{n_i\left(h_i + \sum_{j\neq i} J_{ij} n_j\right)\right\}}{1 + \exp\left\{h_i + \sum_{j\neq i} J_{ij} n_j\right\}} \ , \tag{12}$$

with $\mathbf{n}_{\backslash i} = (n_1, ..., n_{i-1}, n_{i+1}, ..., n_N)$ being the phylogenetic profile of all domains different from i. For a given data PPM, the likelihood with respect to the distribution Eq.(12) – usually called *pseudo-likelihood* –

$$L_i(J_{i,\backslash i}, h_i) = \frac{1}{M}\sum_{i=1}^{M} w_a \log P(n_i^a|\mathbf{n}_{\backslash i}^a) \tag{13}$$

can be easily maximized as a function of $(J_{i,\backslash i}, h_i)$. As is customary in inference problems, we add an ℓ_2 regularization term $\gamma_h \sum_i h_i^2 + \gamma_J \sum_{i\neq j} J_{ij}^2$ to mitigate the effects of overfitting by penalizing parameters with large value. The free parameters γ_J and γ_h are set to $\gamma_J = 0.02$ and $\gamma_h = 0.05$. We refer to [4] for the details of the implementation, which was adapted to binary variables.

C.4 Average product correction (APC)

Another correction, introduced in [13], consists in subtracting from the inferred couplings J_{ij} a contribution due to the single-site properties of i and j (the average over sites is denoted by $\bullet$):

$$J_{ij}^{APC} = J_{ij} - \frac{J_{i\bullet} J_{\bullet j}}{J_{\bullet\bullet}}. \tag{14}$$

Since the APC correction aims to minimize background influences like phylogeny and site entropy, we included it in PhyDCA. While for residue-level DCA, APC-corrected scores have a significantly better prediction accuracy [9], an a posteriori analysis show that the effect is small and almost negligible in PhyDCA (see Figure A).

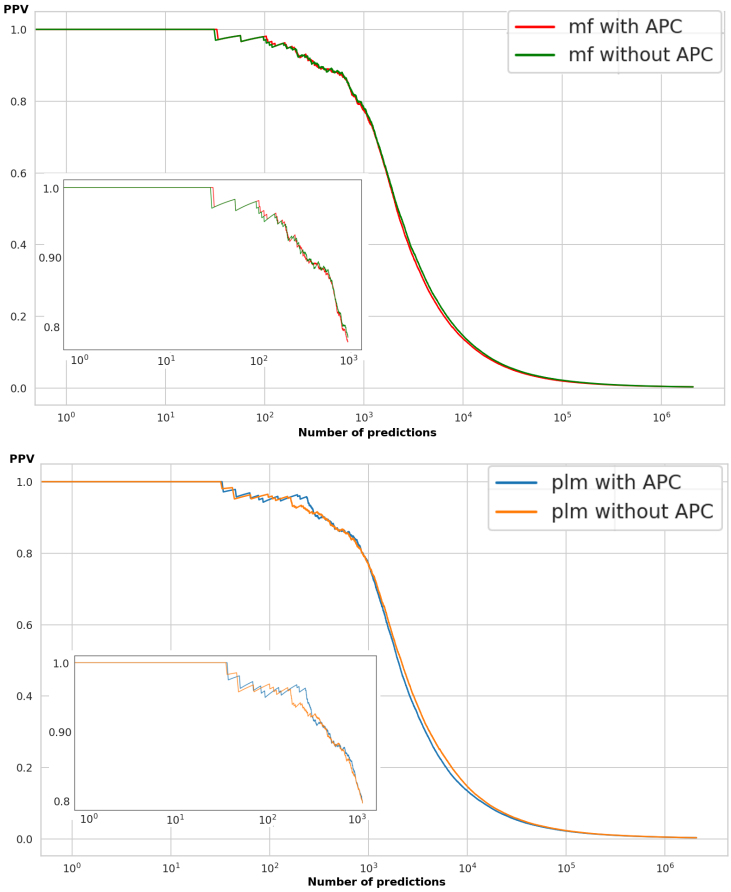

Figure A: **APC correction.** The plots show the PPV curves with and without APC correction for the mean field (upper panel) and pseudo-likelihood (bottom panel) approximations.

D Results

Figure B shows the histograms of the similarity measures (Hamming distance, Pearson correlation, the P-value of the Fisher's exact test and phyletic couplings) for all the pairs of domains considered in the main text. Related domains ought to have profiles of high phyletic couplings, high correlations, low p-value of Fisher's exact test or low Hamming distances, since the first two are similarity, the second two more dissimilarity measures.

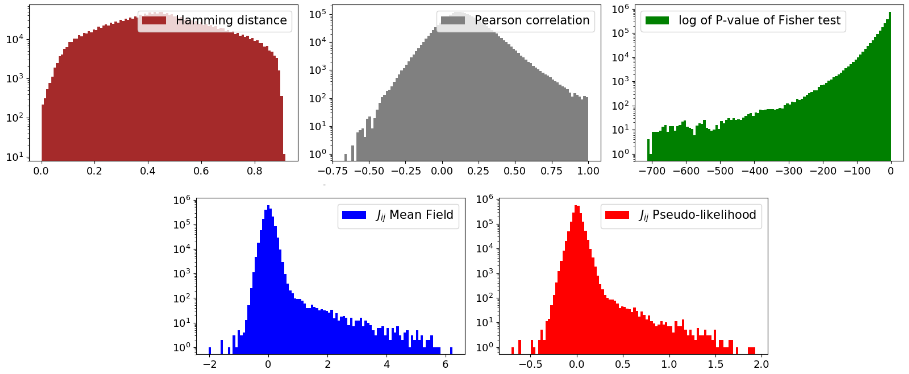

Figure B: **Metrics summary.** The plots show the distribution of the metrics (Hamming distance, Pearson correlation, the P-value of the Fisher's exact test mean-field and pseudo-likelihood phyletic couplings) for all the couples of domains existing in the *E. coli* K-12 MG1655 strain.

In Figure C we plot the phyletic-couplings J_{ij} found using the mean-field (MF) and the pseudo-likelihood maximization (PLM) approximations. Predictions are done by sorting all couplings in decreasing order. They are evidently highly similar, but include a partial reordering. To extract Figure 2 of the main text, the blue dots are interpreted as false positives. From Figure C it is evident that these false positives are – even for very large coupling values – similarly distributed for the two approximations in between the true positives (red points), therefore showing that none of the methods has a clear advantage in precision.

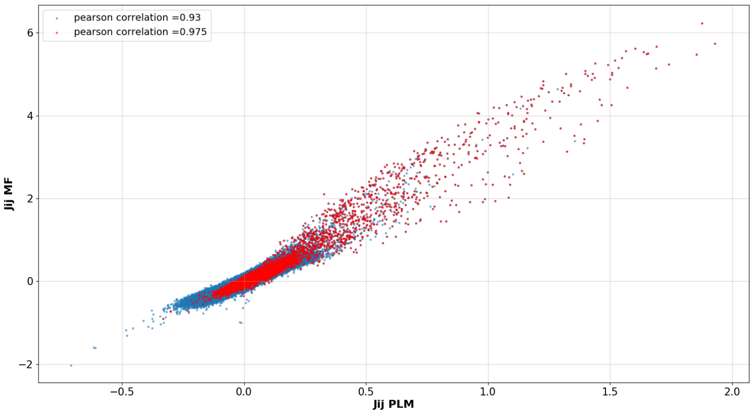

Figure C: **Comparison PLM and MF approximations.** The scatter-plot shows the phyletic couplings found using the MF and the PLM approximations. The blue points are domains pairs are all domain pairs while the red points are those belonging to the positive set of known domain-domain relations (note that the red points form a subset of the blue points). The plot shows that the advantage of PLM over MF (or viceversa) is not visible in the case of domain-domain co-occurrence.

In Figure D we plot the PPV as a function of the couplings (not the cumulative PPV, but PPV per bin of coupling values). It shows that the enrichment of true positive predictions is very high in the tail of large couplings ($J_{plm} > 0.5$ or $J_{MF} > 1.5$), and remains very limited for smaller couplings ($J_{plm} < 0.3$ or $J_{MF} < 0.5$).

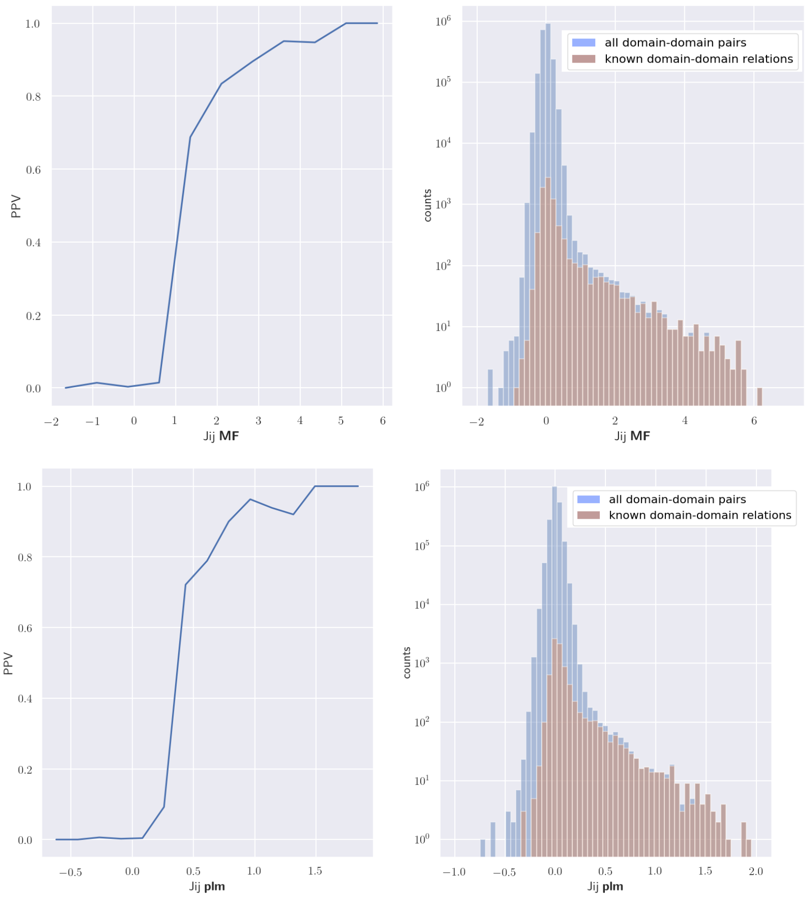

Figure D: **PPV as a function of couplings.** The left figures show the sharp drop of PPV from values close to one for $J_{plm} > 0.5$ with the PLM approximation (or $J_{MF} > 1.5$ in the MF approximation) to very low PPV for $J_{plm} < 0.3$ (or $J_{MF} < 0.5$). The right histograms show the distribution of the phyletic couplings for all domains pairs and for known domain-domain relations. The kink in the histograms between the bulk of small J_{ij} and the tail of large J_{ij} is observed to provide a good cutoff value for high-quality predictions. Once again, it is located close to $J_{plm} = 0.5$ or ($J_{MF} = 1.5$).

E Progressive paralog matching procedure

As discussed in the main text, a large positive phyletic coupling is a strong indicator of a functional relationship between two domains, but not necessarily of a direct physical interaction. To identify physical interactions we use the procedure introduced in [17], which studies coevolution of domain pairs at the

level of the individual residues. We briefly describe the method here; for a full description and additional details we refer to the original publication [17].

Given a pair of strongly coupled domains and the corresponding multiple sequence alignments (say MSA_1 and MSA_2) the problem is to generate a concatenated alignment: we need to find for any sequences belonging to MSA_1 a matching partner in MSA_2. Only sequences belonging to the same species should be matched. This problem has a trivial solution (which we call *matching by uniqueness*) when there are no paralogs and each species has only one sequence in each MSA. However, many protein domains exist in multiple paralogs across many species. In this case, the method proposed in [17] is based on the idea that the correct matching of interacting paralogs should maximize the inter-domain coevolutionary signal. The matched MSA is than used to identify interacting protein families: an average of the four highest inter-protein residue-residue plmDCA scores larger than 0.2 is a strong indicator for a potential interaction, at least of the joint MSA has an effective size $M_{eff} > 200$.

The following procedure was used to create the matched-alignment and compute the DCA scores:

1. download the two PFAM alignments (MSA_1 and MSA_2);
2. if considering a phylogenetic profile matrix with bacterial [eukaryotic] genomes, select only bacterial [eukaryotic] sequences;
3. run the *progressive paralog-matching (PPM)* to find a locally optimal matching;
4. run plmDCA and compute the DCA score, given by the average of the first four highest interdomain residue-residue plmDCA scores.

In Tables A and B we report the first 100 strongly phyletically coupled domain pairs inside the *E. coli* K-12 MG1655 strain *not* belonging to our list of positive known domain-domain relations (i.e. our predictions for such relations).

Figure E and Figure F show the results of the matching procedure for the domain pairs inside the *E. coli* K-12 MG1655 strain.

	pfam ACC dom1	pfam ACC dom2	Phyletic coupling (plm)	Meff joint MSA	paralogs matching DCA score	domain1 description	domain2 description
1	PF03354	PF04860	1.356	203.289	.0733	Phage Terminase	Phage portal protein
2	PF02669	PF03814	1.284	320.623	.3667	K+-transporting ATPase, c chain	Potassium-transporting ATPase A subunit
3	PF02424	PF04205	1.161	457.627	.1663	ApbE family	FMN-binding domain
4	PF00950	PF01297	1.131	480.452	.1813	ABC 3 transport family	Zinc-uptake complex component A periplasmic
5	PF04865	PF04965	1.101	240.503	.1236	Baseplate J-like protein	Gene 25-like lysozyme
6	PF02669	PF02702	0.975	386.368	.1998	K+-transporting ATPase, c chain	Osmosensitive K+ channel His kinase sensor domain
7	PF02702	PF03814	0.951	285.446	.1658	Osmosensitive K+ channel His kinase sensor domain	Potassium-transporting ATPase A subunit
8	PF05930	PF13356	0.779	466.487	.2148	Prophage CP4-57 regulatory protein (AlpA)	Domain of unknown function (DUF4102)
9	PF03972	PF13714	0.763	340.147	.1007	MmgE/PrpD family	Phosphoenolpyruvate phosphomutase
10	PF02614	PF03786	0.758	272.834	.1247	Glucuronate isomerase	D-mannonate dehydratase (UxuA)
11	PF04293	PF06798	0.711	87.4854	.1037	SpoVR like protein	PrkA serine protein kinase C-terminal domain
12	PF04293	PF08298	0.708	92.2474	.1101	SpoVR like protein	PrkA AAA domain
13	PF01924	PF07503	0.707	315.084	.1462	Hydrogenase formation hypA family	HypF finger
14	PF00393	PF02781	0.706	367.212	.1170	6-phosphogluconate dehydrogenase, C-terminal domain	Glucose-6-phosphate dehydrogenase, C-terminal domain
15	PF00393	PF00479	0.706	260.618	.1197	6-phosphogluconate dehydrogenase, C-terminal domain	Glucose-6-phosphate dehydrogenase, NAD binding domain
16	PF04285	PF04293	0.702	65.1382	.1168	Protein of unknown function (DUF444)	SpoVR like protein
17	PF06508	PF14489	0.702	259.401	.1893	Queuosine biosynthesis protein QueC	QueF-like protein
18	PF09344	PF09481	0.695	126.589	.0745	CT1975-like protein	CRISPR-associated protein Cse1 (CRISPR_cse1)
19	PF04965	PF05638	0.687	157.385	.1427	like lysozyme	Type VI secretion system effector, Hcp
20	PF09344	PF09485	0.682	147.077	.1246	CT1975-like protein	CRISPR-associated protein Cse2 (CRISPR_cse2)
21	PF01729	PF02445	0.673	467.581	.1499	Quinolinate phosphoribosyl transferase, C-terminal domain	Quinolinate synthetase A protein
22	PF04865	PF10076	0.670	148.469	.1651	Baseplate J-like protein	Uncharacterised protein conserved in bacteria (DUF2313)
23	PF02601	PF02609	0.668	784.757	.2169	Exonuclease VII, large subunit	Exonuclease VII small subunit
24	PF08798	PF09481	0.666	124.257	.0758	CRISPR associated protein	CRISPR-associated protein Cse1 (CRISPR_cse1)
25	PF01455	PF07503	0.663	407.833	.2654	HupF/HypC family	HypF finger
26	PF00374	PF01924	0.660	138.839	.1017	Nickel-dependent hydrogenase	Hydrogenase formation hypA family
27	PF08798	PF09485	0.655	125.129	.1018	CRISPR associated protein	CRISPR-associated protein Cse2 (CRISPR_cse2)
28	PF02445	PF02749	0.653	448.747	.1698	Quinolinate synthetase A protein	Quinolinate phosphoribosyl transferase, N-terminal domain
29	PF01242	PF06508	0.650	335.013	.1370	6-pyruvoyl tetrahydropterin synthase	Queuosine biosynthesis protein QueC
30	PF02609	PF13742	0.643	615.725	.2052	Exonuclease VII small subunit	OB-fold nucleic acid binding domain
31	PF06750	PF07963	0.641	680.942	.2564	Bacterial Peptidase A24 N-terminal domain	Prokaryotic N-terminal methylation motif
32	PF03239	PF13473	0.636	150.640	.1057	Iron permease FTR1 family	Cupredoxin-like domain
33	PF06968	PF13500	0.632	373.545	.1307	Biotin and Thiamin Synthesis associated domain	AAA domain
34	PF03239	PF09375	0.620	171.344	.0830	Iron permease FTR1 family	Imelysin
35	PF07012	PF10614	0.612	58.7097	.1848	Curlin associated repeat	Type VIII secretion system (T8SS), CsgF protein
36	PF05157	PF06750	0.603	496.496	.2043	Type II secretion system (T2SS), protein E, N-terminal domain	Bacterial Peptidase A24 N-terminal domain
37	PF11739	PF13617	0.597	71.1033	.1694	Dicarboxylate transport	YnbE-like lipoprotein
38	PF00665	PF01527	0.596	503.573	.1382	Integrase core domain	Transposase
39	PF00925	PF00926	0.592	706.081	.1701	GTP cyclohydrolase II	3,4-dihydroxy-2-butanone 4-phosphate synthase
40	PF00374	PF07503	0.588	121.342	.1490	Nickel-dependent hydrogenase	HypF finger
41	PF08279	PF13280	0.579	780.729	.1713	HTH domain	WYL domain
42	PF02617	PF03588	0.577	425.194	.2271	ATP-dependent Clp protease adaptor protein ClpS	Leucyl/phenylalanyl-tRNA protein transferase
43	PF02805	PF06029	0.574	231.377	.2313	Metal binding domain of Ada	AlkA N-terminal domain
44	PF01750	PF01924	0.568	309.585	.1470	Hydrogenase maturation protease	Hydrogenase formation hypA family
45	PF02446	PF02922	0.566	430.385	.1157	4-alpha-glucanotransferase	Carbohydrate-binding module 48 (Isoamylase N-terminal domain)
46	PF02254	PF02386	0.559	597.357	.1114	TrkA-N domain	Cation transport protein
47	PF02219	PF08267	0.555	308.553	.1105	Methylenetetrahydrofolate reductase	Cobalamin-independent synthase, N-terminal domain
48	PF04246	PF13375	0.550	305.417	.1795	Positive regulator of sigma(E), RseC/MucC	RnfC Barrel sandwich hybrid domain
49	PF02261	PF02548	0.548	519.478	.1736	Aspartate decarboxylase	Ketopantoate hydroxymethyltransferase
50	PF02504	PF02660	0.544	656.787	.1571	Fatty acid synthesis protein	Glycerol-3-phosphate acyltransferase

Table A: The first 100 strongly coupled domain pairs inside the *E. coli* K-12 MG1655 strain *not* belonging to our list of positives

	pfam ACC dom1	pfam ACC dom2	Phyletic coupling (plm)	Meff joint MSA	paralogs matching DCA score	domain1 description	domain2 description
51	PF01155	PF07503	0.543	411.187	.1995	Hydrogenase/urease nickel incorporation, metallochaperone, hypA	HypF finger
52	PF01844	PF03354	0.538	143.531	.0831	HNH endonuclease	Phage Terminase
53	PF05336	PF06134	0.536	73.9312	.1221	L-rhamnose mutarotase	L-rhamnose isomerase (RhaA)
54	PF04166	PF07005	0.533	266.819	.1129	Pyridoxal phosphate biosynthetic protein PdxA	Putative sugar-binding N-terminal domain
55	PF02261	PF02569	0.532	678.229	.1897	Aspartate decarboxylase	Pantoate-beta-alanine ligase
56	PF04166	PF17042	0.531	279.134	.1185	Pyridoxal phosphate biosynthetic protein PdxA	Putative nucleotide-binding of sugar-metabolising enzyme
57	PF04349	PF13632	0.528	177.038	.0944	Periplasmic glucan biosynthesis protein, MdoG	Glycosyl transferase family group 2
58	PF09485	PF09707	0.526	109.756	.1593	CRISPR-associated protein Cse2 (CRISPR_cse2)	CRISPR-associated protein (Cas_Cas2CT1978)
59	PF00764	PF14698	0.523	249.501	.1703	Arginosuccinate synthase	Argininosuccinate lyase C-terminal
60	PF00677	PF00885	0.522	665.871	.1768	Lumazine binding domain	6,7-dimethyl-8-ribityllumazine synthase
61	PF01075	PF13580	0.522	449.200	.1101	Glycosyltransferase family 9 (heptosyltransferase)	SIS domain
62	PF00176	PF04434	0.517	450.025	.1183	SNF2 family N-terminal domain	SWIM zinc finger
63	PF00677	PF00926	0.513	801.820	.1766	Lumazine binding domain	3,4-dihydroxy-2-butanone 4-phosphate synthase
64	PF00908	PF16363	0.509	701.521	.1193	dTDP-4-dehydrorhamnose 3,5-epimerase	GDP-mannose 4,6 dehydratase
65	PF09481	PF09707	0.508	78.0705	.0802	CRISPR-associated protein Cse1 (CRISPR_cse1)	CRISPR-associated protein (Cas_Cas2CT1978)
66	PF00885	PF00925	0.506	561.098	.1615	6,7-dimethyl-8-ribityllumazine synthase	GTP cyclohydrolase II
67	PF07027	PF11739	0.504	78.5197	.1112	Protein of unknown function (DUF1318)	Dicarboxylate transport
68	PF01750	PF07503	0.504	308.601	.2008	Hydrogenase maturation protease	HypF finger
69	PF09481	PF09704	0.498	112.612	.0737	CRISPR-associated protein Cse1 (CRISPR_cse1)	CRISPR-associated protein (Cas_Cas5)
70	PF00885	PF00926	0.497	590.538	.2166	6,7-dimethyl-8-ribityllumazine synthase	3,4-dihydroxy-2-butanone 4-phosphate synthase
71	PF00677	PF00925	0.494	757.052	.1534	Lumazine binding domain	GTP cyclohydrolase II
72	PF03773	PF13192	0.484	132.523	.1292	Predicted permease	Thioredoxin domain
73	PF00468	PF01783	0.483	407.211	.3312	Ribosomal protein L34	Ribosomal L32p protein family
74	PF04228	PF05872	0.481	267.316	.1161	Putative neutral zinc metallopeptidase	Bacterial protein of unknown function (DUF853)
75	PF01227	PF02152	0.476	496.776	.1692	GTP cyclohydrolase I	Dihydroneopterin aldolase
76	PF00370	PF16901	0.461	421.729	.1283	FGGY family of carbohydrate kinases, N-terminal domain	C-terminal domain of alpha-glycerophosphate oxidase
77	PF02782	PF16901	0.459	394.171	.1453	FGGY family of carbohydrate kinases, C-terminal domain	C-terminal domain of alpha-glycerophosphate oxidase
78	PF01924	PF14720 0.	5853	222.024	.1429	ase formation hypA family	NiFe/NiFeSe hydrogenase small subunit C-terminal
79	PF00444	PF00468	0.453	228.453	.3186	Ribosomal protein L36	Ribosomal protein L34
80	PF13742	PF17191	0.452	632.988	.1555	OB-fold nucleic acid binding domain	RecG wedge domain
81	PF01326	PF03618	0.451	103.633	.1298	Pyruvate phosphate dikinase, PEP/pyruvate binding domain	Kinase/pyrophosphorylase
82	PF02283	PF02572	0.450	468.079	.1683	Cobinamide kinase / cobinamide phosphate guanyltransferase	ATP:corrinoid adenosyltransferase BtuR/CobO/CobP
83	PF04973	PF13521	0.450	169.274	.1138	Nicotinamide mononucleotide transporter	AAA domain
84	PF04257	PF13538	0.448	134.063	.0906	Exodeoxyribonuclease V, gamma subunit	UvrD-like helicase C-terminal domain
85	PF09485	PF09704	0.447	150.926	.1319	ssociated protein Cse2 (CRISPR_cse2)	CRISPR-associated protein (Cas_Cas5)
86	PF00128	PF02446	0.444	351.543	.1027	Alpha amylase, catalytic domain	4-alpha-glucanotransferase
87	PF04333	PF05494	0.440	296.656	.1366	MlaA lipoprotein	MlaC protein
88	PF04865	PF07484	0.432	168.580	.1083	Baseplate J-like protein	Phage Tail Collar Domain
89	PF13091	PF13396	0.428	514.594	.1711	PLD-like domain	Phospholipase_D-nuclease N-terminal
90	PF02675	PF03994	0.424	132.522	.1567	S-adenosylmethionine decarboxylase	Domain of Unknown Function (DUF350)
91	PF00926	PF01872	0.423	888.552	.1667	3,4-dihydroxy-2-butanone 4-phosphate synthase	RibD C-terminal domain
92	PF02446	PF02806	0.422	405.848	.1156	4-alpha-glucanotransferase	Alpha amylase, C-terminal all-beta domain
93	PF07503	PF14720	0.420	132.635	.2865	HypF finger	NiFe/NiFeSe hydrogenase small subunit C-terminal
94	PF02568	PF08349	0.419	128.183	.1489	Thiamine biosynthesis protein (ThiI)	Protein of unknown function (DUF1722)
95	PF03783	PF07012	0.419	56.7289	.1223	Curli production assembly/transport component CsgG	Curlin associated repeat
96	PF03453	PF06463	0.418	752.101	.2050	MoeA N-terminal region (domain I and II)	Molybdenum Cofactor Synthesis C
97	PF08402	PF13343	0.418	547.287	.1188	TOBE domain	Bacterial extracellular solute-binding protein
98	PF01967	PF03453	0.418	859.202	.2267	MoaC family	MoeA N-terminal region (domain I and II)
99	PF02401	PF04551	0.414	476.398	.1403	LytB protein	GcpE protein
100	PF00925	PF01872	0.414	780.584	.1436	GTP cyclohydrolase II	RibD C-terminal domain
101	PF02589	PF02652	0.412	111.010	.0974	LUD domain	L-lactate permease

Table B: The first 100 strongly coupled domain pairs inside the *E. coli* K-12 MG1655 strain *not* belonging to our list of positives

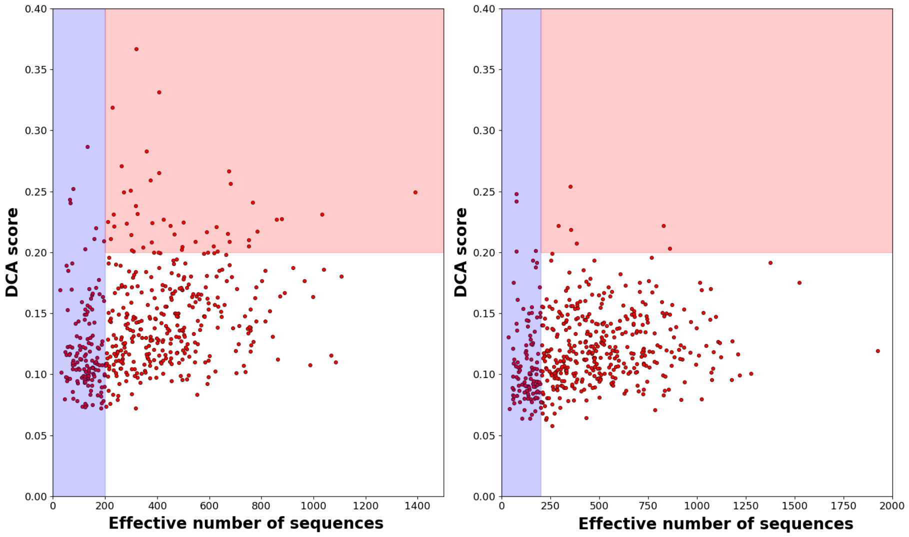

Figure E: **Matching procedure for *E. coli*.** The panel on the left shows the result of the matching procedure for the 500 most significant predictions for domain families existing inside the K12 strain of *E. coli* (the list can be found on the `Github` page at `results/ECOLI_matching_results.dat`). On the right, as a comparison, a random matching for the same domain pairs.

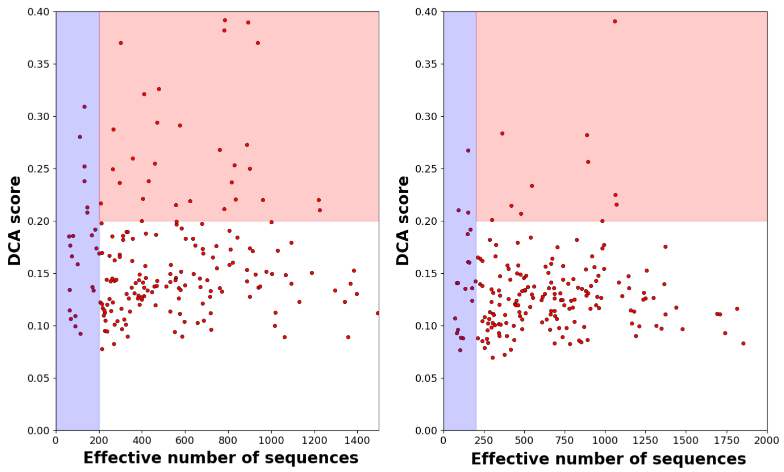

Figure F: **Matching procedure for *E. coli.* domains in iPfam** The panel on the left shows the result of the matching procedure for the 200 pairs of highest phylogenetic couplings belonging to the iPfam database. The complete list can be found on the `Github` page at `results/ECOLI_matching_iPfam_results.dat` . On the right, as a comparison, a random matching for the same domain pairs.

Figure G show the results of residue-level DCA for the 200 domain pairs of strongest phyletic couplings, which are co-localized in one protein in E. coli. Due to the co-localization, the generation of a joint MSA is trivial in this case; the paralogs-matching can be avoided. Note that domains can co-occur in the same protein without direct physical interactions. Out of the 200 pairs, 144 of these domain pairs are also listed in iPfam, meaning that a direct physical interaction is structurally known.

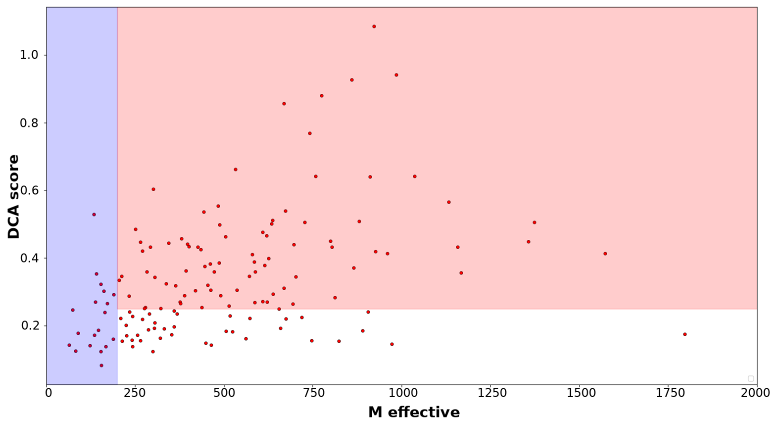

Figure G: **Results of residue-level DCA for domains co-localized in the same protein.** The panel shows the results of residue-level DCA for the 200 domain pairs of strongest phyletic couplings. Co-localization does not necessarily imply physical interaction. However, out of 200 pairs, 144 are also listed in iPfam database as being in physical contact in experimentally resolved PDB structures. Due to their co-localization, the generation of a joint MSA is trivial in this case and the paralogs-matching can be avoided.

E.1 Network analysis

As stated in the main text, CoPAP and PhyDCA treat very different confounding factors of coevolutionary analysis – phylogenetic biases and indirect correlations. Nevertheless from Figure 3 of the main text, it appears that almost none of the correlated pairs strongly coupled in PhyDCA, are actually discarded by CoPAP. But are the correlations of pairs, which are retained by CoPAP as non phyletically coupled, but discarded by PhyDCA, really an indirect network effect of the PhyDCA couplings?

To answer this question, we first introduce in Fig. H two scatter plots of the phyletic couplings vs. Pearson correlations between domain pairs, in the first case for the 3611 domain pairs of highest CoPAP score, in the second case for all domain pairs. In both cases, we see a clear triangular shape, indicating that large couplings lead to large correlations, but large correlations can exist between weakly coupled pairs. Since our PhyDCA model reproduces correlations using couplings, the latter case must result from indirect correlations. Also as a consequence, the phyletic coupling network is substantially sparser than the correlation network.

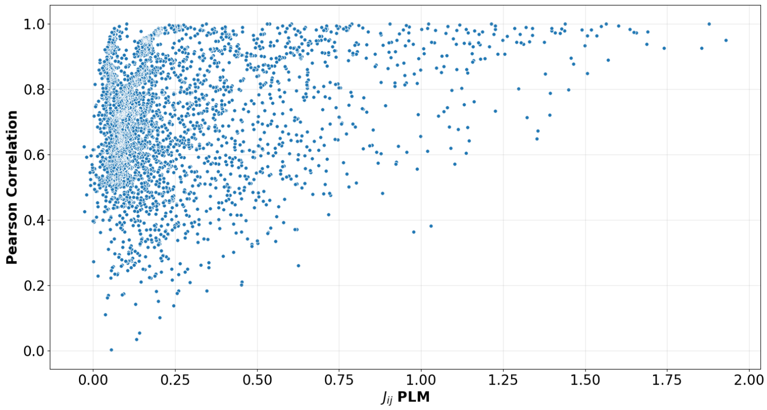

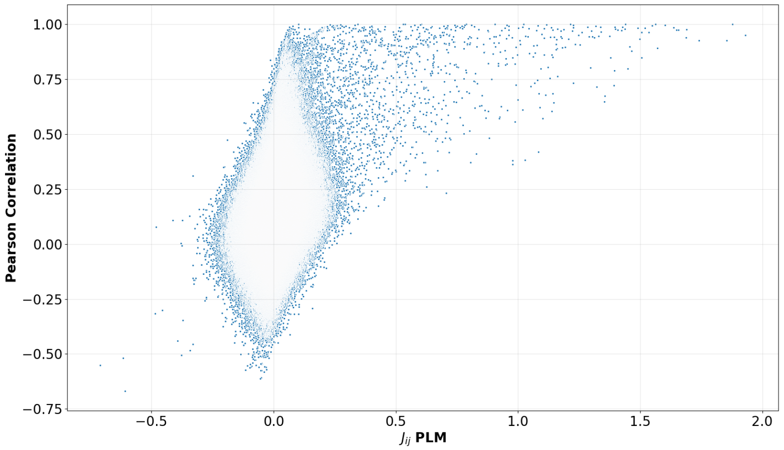

Figure H: **Couplings vs. correlations.** The figure shows a scatter plot of PhyDCA couplings vs. Pearson correlations, for the 3611 domain pairs of highest CoPAP score in the upper panel, and for all domain pairs in the lower one.

To corroborate this, in Fig. I, we consider the network of the 1000 strongest phyletic couplings and study the correlations as a function of the shortest-path distance between domains along this network. Correlations decrease with distance until they saturate at a low but non-zero level. This is coherent with the idea that empirical correlations found in the data have at least three contributions – direct correlations induced by direct couplings (at distance 1), indirect couplings induced by coupling chains, and a ground level of correlations, which possibly result from phylogenetic correlations between the species and other sampling effects.

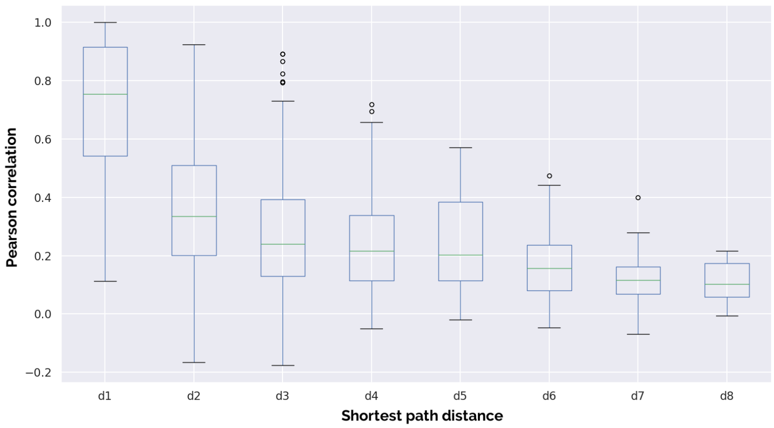

Figure I: **Correlation decay on PhyDCA network.** The figure shows the decay of empirical correlations between pairs of domain belonging to the network of the first 1000 strongest phyletic couplings as a function of their shortest-path distance on this network.

If we take alternatively the network induced by the 1613 pairs, which have large Pearson correlations and are preserved by CoPAP (the intersection of the red and green circles in Fig. 3A in the main text), we also find a correlation decrease (as to be expected in any sparsely connected graphical model), cf. Fig. J. However, the decay is slower than on the PhyDCA network, even if the network is denser. Pairs in the PhyDCA network are thus less correlated than pairs at the same distance in the correlation network, which shows that the phyletic coupling network more parsimoniously explains the connectivity patterns present in the data.

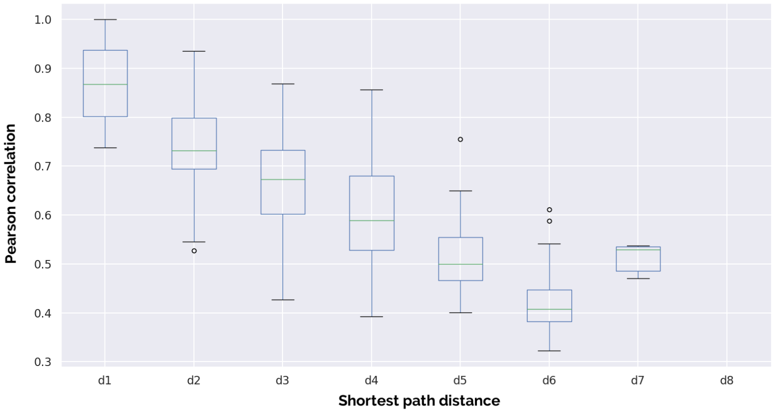

Figure J: **Correlation decay on CoPAP-Pearson network.** The figure shows the decay of empirical correlations between pairs of domain belonging to the network of the 1613 domain pairs of strongest CoPAP-preserved Pearson correlations (the intersection of the red and green circles in Fig. 3A in the main text) as a function of their shortest-path distance on this network.

F All bacteria

In the main text we use the model organism *Escherichia coli* as reference genome in order to have a large set of known domain-domain relationships. In this section we consider a broader selection of genomes by applying the same methodology to all 9,358 Pfam domains appearing in bacteria. To access the accuracy of our prediction we compile a number of known domain-domain relationships: **intra-protein localization** (out of 2,972,104 proteins 866,591 contain multiple domains, giving rise to 26,381 distinct domain-domain relations), **domain-domain contacts in 3d structures** (from the iPfam database, for a total of 545 known relationships), **protein-protein interaction** (from the IntAct database, obtaining 67,409 domain pairs). This leads to a total of 92,428 known relationship (cf. Figure K, Panel A).

We then select the couplings between domains which are only present in *E. coli* genome (cf. Figure K, Panel B and C) finding 96% correlation with the couplings inferred in the main text, thus proving the robustness of the results with respect to the selection of domains.

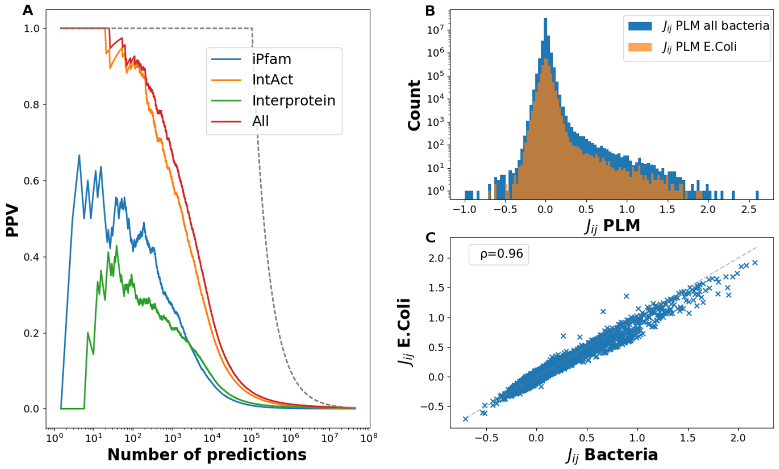

Figure K: **Phylogenetic couplings** Panel A shows the PPV of the phyletic couplings of all bacterial domains for predicting domain-domain relationships (including protein architecture, iPfam and IntAct entries). Panel B shows a histogram of couplings J_{ij}, as inferred by PLM, for the domains present in all bacteria and for those appearing only in *E. coli*. In Panel C we retain from the bacterial phyletic couplings only the couplings between domains present in *E. coli*. Then we compare them with the couplings found by the procedure described in the main text, finding a correlation of 96% between the two.

We have applied the paralog-matching analysis to the 200 most coupled bacterial domain pairs (see Figure L). A list of the domain pairs, their phylogenetic coupling and the DCA score can be found on the `Github` page at `results/ALLBACTERIA_matching_results.dat`).

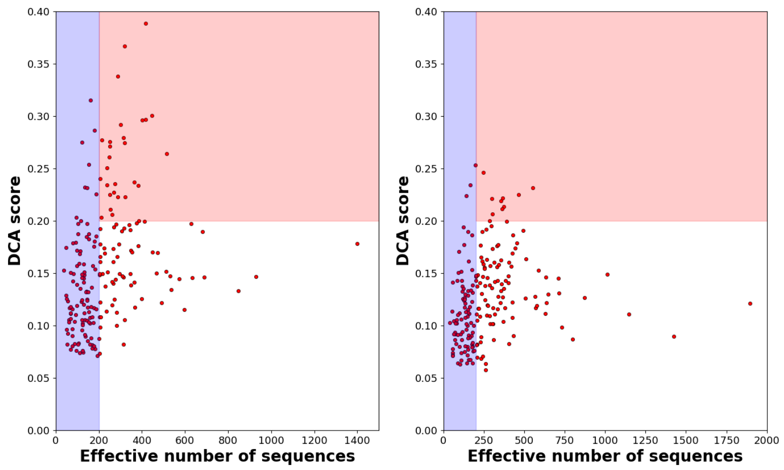

Figure L: **Matching procedure for bacteria:** Panel A shows the effective sequence number and the DCA scores for the 200 most significant PhyDCA predictions. Panel B shows a random matching for the same domain pairs.

F.1 Negative phyletic couplings

As discussed in the main text, a negative phyletic coupling disfavors the joint presence of two domains in the same genome and thereby highlights alternative solutions for the same functionality. In Table D we report the 100 most strongly negatively coupled domain pairs with their PFAM description.

G Eukaryotic genomes: human as reference species

The complete list of results of our analyisis applied on eukaryotic genomes using human as reference species can be found on the `Github` page at `results/HUMAN_matching_results`.

	pfam ACC dom1	pfam ACC dom2	Phyletic coupling	domain1 description	domain2 description
1	PF00303	PF02511	-0.9978	Thymidylate synthase	Thymidylate synthase complementing protein
2	PF01220	PF01487	-0.9277	Dehydroquinase class II	Type I 3-dehydroquinase
3	PF02834	PF13563	-0.9075	LigT like Phosphoesterase	2'-5' RNA ligase superfamily
4	PF00406	PF13207	-0.8258	Adenylate kinase	AAA domain
5	PF01205	PF02594	-0.7077	Uncharacterized protein family UPF0029	Uncharacterised ACR, YggU family COG1872
6	PF13623	PF13624	-0.7051	SurA N-terminal domain	SurA N-terminal domain
7	PF04816	PF12847	-0.6316	tRNA (adenine(22)-N(1))-methyltransferase	Methyltransferase domain
8	PF00636	PF14622	-0.6281	Ribonuclease III domain	Ribonuclease-III-like
9	PF00186	PF02511	-0.6281	Dihydrofolate reductase	Thymidylate synthase complementing protein
10	PF01227	PF02649	-0.6118	GTP cyclohydrolase I	Type I GTP cyclohydrolase folE2
11	PF06745	PF13481	-0.5844	KaiC	AAA domain
12	PF02677	PF08331	-0.581	Uncharacterized BCR, COG1636	Domain of unknown function (DUF1730)
13	PF02696	PF03190	-0.5651	Uncharacterized ACR, YdiU/UPF0061 family	Protein of unknown function, DUF255
14	PF00311	PF02436	-0.5432	Phosphoenolpyruvate carboxylase	Conserved carboxylase domain
15	PF02502	PF06026	-0.5371	Ribose/Galactose Isomerase	Ribose 5-phosphate isomerase A (phosphoriboisomerase A)
16	PF00245	PF05787	-0.5333	Alkaline phosphatase	Bacterial protein of unknown function (DUF839)
17	PF00075	PF13456	-0.5317	RNase H	Reverse transcriptase-like
18	PF01169	PF02659	-0.5294	Uncharacterized protein family UPF0016	Putative manganese efflux pump
19	PF01321	PF05195	-0.5165	Creatinase/Prolidase N-terminal domain	Aminopeptidase P, N-terminal domain
20	PF02594	PF09186	-0.5139	Uncharacterised ACR, YggU family COG1872	Domain of unknown function (DUF1949)
21	PF02595	PF13660	-0.5071	Glycerate kinase family	Domain of unknown function (DUF4147)
22	PF02595	PF05161	-0.4983	Glycerate kinase family	MOFRL family
23	PF00491	PF04371	-0.4956	Arginase family	Porphyromonas-type peptidyl-arginine deiminase
24	PF02664	PF05221	-0.4583	S-Ribosylhomocysteinase (LuxS)	S-adenosyl-L-homocysteine hydrolase
25	PF00719	PF02833	-0.453	Inorganic pyrophosphatase	DHHA2 domain
26	PF00670	PF02664	-0.446	S-adenosyl-L-homocysteine hydrolase, NAD binding domain	S-Ribosylhomocysteinase (LuxS)
27	PF04306	PF07264	-0.445	Protein of unknown function (DUF456)	Etoposide-induced protein 2.4 (EI24)
28	PF00141	PF06628	-0.4378	Peroxidase	Catalase-related immune-responsive
29	PF13441	PF13488	-0.4371	YMGG-like Gly-zipper	Glycine zipper
30	PF08328	PF10397	-0.4304	Adenylosuccinate lyase C-terminal	Adenylosuccinate lyase C-terminus
31	PF00821	PF01293	-0.4195	Phosphoenolpyruvate carboxykinase	Phosphoenolpyruvate carboxykinase
32	PF03100	PF05140	-0.4192	CcmE	ResB-like family
33	PF01027	PF12811	-0.4075	Inhibitor of apoptosis-promoting Bax1	Bax inhibitor 1 like
34	PF03379	PF05140	-0.4022	CcmB protein	ResB-like family
35	PF01863	PF10263	-0.3981	Protein of unknown function DUF45	SprT-like family
36	PF01458	PF07743	-0.396	Uncharacterized protein family (UPF0051)	HSCB C-terminal oligomerisation domain
37	PF05140	PF16327	-0.3919	ResB-like family	Cytochrome c-type biogenesis protein CcmF C-terminal
38	PF01878	PF03883	-0.3849	EVE domain	Peroxide stress protein YaaA
39	PF03352	PF08713	-0.3776	Methyladenine glycosylase	DNA alkylation repair enzyme
40	PF01592	PF02657	-0.3774	NifU-like N terminal domain	Fe-S metabolism associated domain
41	PF04011	PF13421	-0.3727	LemA family	SPFH domain-Band 7 family
42	PF00274	PF01116	-0.3667	Fructose-bisphosphate aldolase class-I	Fructose-bisphosphate aldolase class-II
43	PF01817	PF07736	-0.364	Chorismate mutase type II	Chorismate mutase type I
44	PF01070	PF02589	-0.3613	FMN-dependent dehydrogenase	LUD domain
45	PF01943	PF13440	-0.3613	Polysaccharide biosynthesis protein	Polysaccharide biosynthesis protein
46	PF00282	PF13086	-0.355	Pyridoxal-dependent decarboxylase conserved domain	AAA domain
47	PF00141	PF00199	-0.3527	Peroxidase	Catalase
48	PF01458	PF01491	-0.3511	Uncharacterized protein family (UPF0051)	Frataxin-like domain
49	PF00368	PF02542	-0.3461	Hydroxymethylglutaryl-coenzyme A reductase	YgbB family

Table C: The 100 most strongly negative coupled domain pairs. Negative phyletic couplings identify alternative solutions for the same functionality.

	pfam ACC dom1	pfam ACC dom2	Phyletic coupling	domain1 description	domain2 description
50	PF01523	PF08367	-0.3405	Putative modulator of DNA gyrase	Peptidase M16C associated
51	PF02386	PF02705	-0.3401	Cation transport protein	K+ potassium transporter
52	PF00368	PF04551	-0.3398	Hydroxymethylglutaryl-coenzyme A reductase	GcpE protein
53	PF00368	PF02401	-0.3385	Hydroxymethylglutaryl-coenzyme A reductase	LytB protein
54	PF01977	PF16582	-0.3343	3-octaprenyl-4-hydroxybenzoate carboxy-lyase	Middle domain of thiamine pyrophosphate
55	PF13476	PF13514	-0.3331	AAA domain	AAA domain
56	PF00368	PF01128	-0.3326	Hydroxymethylglutaryl-coenzyme A reductase	2-C-methyl-D-erythritol 4-phosphate cytidylyltransferase
57	PF04204	PF07021	-0.3316	Homoserine O-succinyltransferase	Methionine biosynthesis protein MetW
58	PF00145	PF11907	-0.3261	C-5 cytosine-specific DNA methylase	Domain of unknown function (DUF3427)
59	PF01458	PF04384	-0.326	Uncharacterized protein family (UPF0051)	Iron-sulphur cluster assembly
60	PF01699	PF03741	-0.3253	Sodium/calcium exchanger protein	Integral membrane protein TerC family
61	PF01797	PF13087	-0.3217	Transposase IS200 like	AAA domain
62	PF02675	PF16653	-0.3216	S-adenosylmethionine decarboxylase	Saccharopine dehydrogenase C-terminal domain
63	PF04402	PF13598	-0.3197	Protein of unknown function (DUF541)	Domain of unknown function (DUF4139)
64	PF01544	PF01769	-0.3186	CorA-like Mg2+ transporter protein	Divalent cation transporter
65	PF13145	PF13616	-0.3184	PPIC-type PPIASE domain	PPIC-type PPIASE domain
66	PF01070	PF11870	-0.3179	FMN-dependent dehydrogenase	Domain of unknown function (DUF3390)
67	PF01904	PF03649	-0.3159	Protein of unknown function DUF72	Uncharacterised protein family (UPF0014)
68	PF01226	PF04657	-0.3145	Formate/nitrite transporter	Putative inner membrane exporter, YdcZ
69	PF03547	PF13579	-0.3136	Membrane transport protein	Glycosyl transferase 4-like domain
70	PF09992	PF13276	-0.3116	Phosphodiester glycosidase	HTH-like domain
71	PF00444	PF00872	-0.3092	Ribosomal protein L36	Transposase, Mutator family
72	PF01070	PF06127	-0.3081	FMN-dependent dehydrogenase	Protein of unknown function (DUF962)
73	PF00444	PF13338	-0.3052	Ribosomal protein L36	Transcriptional regulator, AbiEi antitoxin
74	PF00368	PF02670	-0.3052	Hydroxymethylglutaryl-coenzyme A reductase	1-deoxy-D-xylulose 5-phosphate reductoisomerase
75	PF00368	PF08436	-0.3044	Hydroxymethylglutaryl-coenzyme A reductase	1-deoxy-D-xylulose 5-phosphate reductoisomerase C-terminal
76	PF00368	PF13288	-0.3044	Hydroxymethylglutaryl-coenzyme A reductase	DXP reductoisomerase C-terminal domain
77	PF01458	PF01592	-0.3038	Uncharacterized protein family (UPF0051)	NifU-like N terminal domain
78	PF14789	PF14805	-0.3036	Tetrahydrodipicolinate N-succinyltransferase middle	Tetrahydrodipicolinate N-succinyltransferase N-terminal
79	PF01042	PF14588	-0.3036	Endoribonuclease L-PSP	YjgF/chorismate_mutase-like, putative endoribonuclease
80	PF02110	PF05690	-0.302	Hydroxyethylthiazole kinase family	Thiazole biosynthesis protein ThiG
81	PF02677	PF13484	-0.3017	Uncharacterized BCR, COG1636	4Fe-4S double cluster binding domain
82	PF13414	PF13738	-0.3002	TPR repeat	Pyridine nucleotide-disulphide oxidoreductase
83	PF03073	PF08212	-0.2999	TspO/MBR family	Lipocalin-like domain
84	PF03592	PF13671	-0.2998	Terminase small subunit	AAA domain
85	PF01244	PF07784	-0.2997	Membrane dipeptidase (Peptidase family M19)	Protein of unknown function (DUF1622)
86	PF02900	PF13384	-0.2991	Catalytic LigB subunit of aromatic ring-opening dioxygenase	Homeodomain-like domain
87	PF01391	PF05050	-0.2976	Collagen triple helix repeat (20 copies)	Methyltransferase FkbM domain
88	PF03413	PF13936	-0.2971	Peptidase propeptide and YPEB domain	Helix-turn-helix domain
89	PF01988	PF16916	-0.2956	VIT family	Dimerisation domain of Zinc Transporter
90	PF08309	PF13088	-0.2952	LVIVD repeat	BNR repeat-like domain
91	PF00317	PF08471	-0.2947	Ribonucleotide reductase, all-alpha domain	Class II vitamin B12-dependent ribonucleotide reductase
92	PF11127	PF14524	-0.2947	Protein of unknown function (DUF2892)	Wzt C-terminal domain
93	PF00565	PF07394	-0.2945	Staphylococcal nuclease homologue	Protein of unknown function (DUF1501)
94	PF09954	PF12844	-0.2938	Uncharacterized protein conserved in bacteria (DUF2188)	Helix-turn-helix domain
95	PF02635	PF13440	-0.293	DsrE/DsrF-like family	Polysaccharide biosynthesis protein
96	PF01906	PF01987	-0.2929	Putative heavy-metal-binding	Mitochondrial biogenesis AIM24
97	PF07582	PF13492	-0.2927	AP endonuclease family 2 C terminus	GAF domain
98	PF02230	PF13740	-0.2915	Phospholipase/Carboxylesterase	ACT domain
99	PF02535	PF13274	-0.2907	ZIP Zinc transporter	Protein of unknown function (DUF4065)
100	PF01680	PF03740	-0.2907	SOR/SNZ family	SOR/SNZ family

Table D: The 100 most strongly negative coupled domain pairs. Negative phyletic couplings identify alternative solutions for the same functionality.

References

[1] The UniProt Consortium. Uniprot: a hub for protein information. Nucleic Acids Research, 43(D1):D204–D212, 2015.

[2] Bateman, A., Coin, L., Durbin, R., Finn, R.D., Hollich, V., Griffiths-Jones, S., Khanna, A., Marshall, M., Moxon, S., Sonnhammer, E.L. and Studholme, D.J., 2004. The Pfam protein families database. Nucleic acids research, 32(suppl 1), pp.D138-D141.

[3] De Las Rivas, J., Fontanillo, C. (2010). Protein–protein interactions essentials: key concepts to building and analyzing interactome networks. PLoS Comput Biol, 6(6), e1000807.

[4] M. Ekeberg, C. Lovkvist, Y. Lan, M. Weigt, and E. Aurell. Improved contact prediction in proteins: Using pseudolikelihoods to infer potts models. Physical Review E, 87(1):012707, 2013.

[5] Pellegrini, M., Marcotte, E. M., Thompson, M. J., Eisenberg, D., Yeates, T. O. (1999). Assigning protein functions by comparative genome analysis: protein phylogenetic profiles. Proceedings of the National Academy of Sciences, 96(8), 4285-4288.

[6] Harrington, E. D., Jensen, L. J., Bork, P. (2008). Predicting biological networks from genomic data. FEBS letters, 582(8), 1251-1258.

[7] Bateman, A., Coin, L., Durbin, R., Finn, R. D., Hollich, V., Griffiths-Jones, S., Studholme, D. J. (2004). The Pfam protein families database. Nucleic acids research, 32(suppl 1), D138-D141.

[8] Reed, J. L., Vo, T. D., Schilling, C. H., Palsson, B. O. (2003). An expanded genome-scale model of Escherichia coli K-12 (i JR904 GSM/GPR). Genome biology, 4(9), R54.

[9] Cocco, S., Feinauer, C., Figliuzzi, M., Monasson, R. and Weigt, M., 2017. Inverse Statistical Physics of Protein Sequences: A Key Issues Review. arXiv preprint arXiv:1703.01222.

[10] Pagel, P., Wong, P., Frishman, D. (2004). A domain interaction map based on phylogenetic profiling. Journal of molecular biology, 344(5), 1331-1346.

[11] Kensche, P. R., van Noort, V., Dutilh, B. E., Huynen, M. A. (2008). Practical and theoretical advances in predicting the function of a protein by its phylogenetic distribution. Journal of the Royal Society Interface, 5(19), 151-170.

[12] Finn, R. D., Marshall, M., Bateman, A. (2005). iPfam: visualization of protein–protein interactions in PDB at domain and amino acid resolutions. Bioinformatics, 21(3), 410-412.

[13] Dunn, S.D., Wahl, L.M. and Gloor, G.B., 2007. Mutual information without the influence of phylogeny or entropy dramatically improves residue contact prediction. Bioinformatics, 24(3), pp.333-340.

[14] Orchard, S., Ammari, M., Aranda, B., Breuza, L., Briganti, L., Broackes-Carter, F., Duesbury, M. (2013). The MIntAct project—IntAct as a common curation platform for 11 molecular interaction databases. Nucleic acids research, gkt1115.

[15] Jeff Bezanzon, Stefan Karpinski, Viral Shah, and Alan Edelman. Julia: A fast dynamic language for technical computing. In Lang.NEXT, April 2012.

[16] Raghavachari, B., Tasneem, A., Przytycka, T. M., Jothi, R. (2008). DOMINE: a database of protein domain interactions. Nucleic acids research, 36(suppl 1), D656-D661.

[17] Gueudré, T., Baldassi, C., Zamparo, M., Weigt, M., Pagnani, A. (2016). Simultaneous identification of specifically interacting paralogs and inter-protein contacts by direct coupling analysis. Proceedings of the National Academy of Sciences, 113(43), 12186-12191.
